# Faulty autolysosome acidification in Alzheimer’s disease mouse models induces autophagic build-up of Aβ in neurons, yielding senile plaques

**DOI:** 10.1038/s41593-022-01084-8

**Published:** 2022-06-02

**Authors:** Ju-Hyun Lee, Dun-Sheng Yang, Chris N. Goulbourne, Eunju Im, Philip Stavrides, Anna Pensalfini, Han Chan, Cedric Bouchet-Marquis, Cynthia Bleiwas, Martin J. Berg, Chunfeng Huo, James Peddy, Monika Pawlik, Efrat Levy, Mala Rao, Mathias Staufenbiel, Ralph A. Nixon

**Affiliations:** 1 Center for Dementia Research, Nathan S. Kline Institute, Orangeburg, NY USA; 2 Department of Psychiatry, New York University Langone Health, New York, NY USA; 3 Thermo Fisher Scientific, Hillsboro, OR USA; 4 Departments of Biochemistry & Molecular Pharmacology, New York University Langone Health, New York, NY USA; 5 NYU Neuroscience Institute, New York University Langone Health, New York, NY USA; 6 Department of Cellular Neurology, Hertie Institute for Clinical Brain Research, University of Tübingen, Tübingen, Germany; 7 Department of Cell Biology, New York University Langone Health, New York, NY USA

**Keywords:** Alzheimer's disease, Autophagy, Lysosomes, Cellular neuroscience

## Abstract

Autophagy is markedly impaired in Alzheimer’s disease (AD). Here we reveal unique autophagy dysregulation within neurons in five AD mouse models in vivo and identify its basis using a neuron-specific transgenic mRFP-eGFP-LC3 probe of autophagy and pH, multiplex confocal imaging and correlative light electron microscopy. Autolysosome acidification declines in neurons well before extracellular amyloid deposition, associated with markedly lowered vATPase activity and build-up of Aβ/APP-βCTF selectively within enlarged de-acidified autolysosomes. In more compromised yet still intact neurons, profuse Aβ-positive autophagic vacuoles (AVs) pack into large membrane blebs forming flower-like perikaryal rosettes. This unique pattern, termed PANTHOS (poisonous anthos (flower)), is also present in AD brains. Additional AVs coalesce into peri-nuclear networks of membrane tubules where fibrillar β-amyloid accumulates intraluminally. Lysosomal membrane permeabilization, cathepsin release and lysosomal cell death ensue, accompanied by microglial invasion. Quantitative analyses confirm that individual neurons exhibiting PANTHOS are the principal source of senile plaques in amyloid precursor protein AD models.

## Abstract (web summary)

Interrogation of neuronal autophagy in vivo in Alzheimerʼs disease mouse models identified deficient autolysosome acidification as the basis for extreme autophagic stress, yielding β-amyloid accumulation within intact neurons, which are the main source of senile plaques.

## Main

Autophagy is the principal pathway for lysosomal degradation, maintaining cellular homeostasis by constitutively turning over obsolete proteins and organelles. It is induced further by disease and cell stress to eliminate abnormal proteins, aggregates and damaged organelles^1–4^. Autophagy encompasses several mechanisms for sequestering substrates and their delivery to lysosomes (LYs). In the major autophagy–lysosomal pathway (ALP), macroautophagy, an elongating double membrane envelops cytoplasm or, via adaptor protein, engages specific targeted substrates and then closes to form an autophagosome (AP). APs mature to autolysosomes (ALs) upon fusion with LY or endolysosome, which introduces varied cathepsin proteases, other acid hydrolases and vATPase, the proton pump that acidifies AL lumens and activates the hydrolases. LYs are targets of causative gene products and risk factors for AD^5^, including the pathogenic amyloid precursor protein (APP) metabolites APP-βCTF and Aβ^6^ that are actively generated within endosomal and autophagic pathways and are normally cleared by LYs^7^.

AD is defined neuropathologically by two lesions: intracellular tau aggregates (neurofibrillary tangles) and neuritic plaques composed of focally swollen (dystrophic) neurites (DNs)^8^, extracellular β-amyloid and many other proteins^9^. Additionally, AVs containing incompletely digested autophagy substrates accumulate progressively within affected neurons at the earliest disease stage^5,10–12^. The molecular basis for autophagy dysfunction in AD, its relationship to APP/amyloid pathology and its pathogenic implications are unclear due, in part, to technical challenges of monitoring ALP abnormalities in vivo in brain. To overcome these limitations, we generated transgenic mice (TRGL) with neuron-specific expression of tandem fluorescence-tagged LC3 (mRFP-eGFP-LC3 or tfLC3), an autophagy adaptor protein selectively associated with AP and AL^13^. The tfLC3 probe enabled us to investigate individual vesicular components of the neuronal ALP in intact brain and, to our knowledge for the first time, assess AL acidification ratiometrically in neurons throughout disease progression in mouse AD models.

To identify and monitor AD-related ALP deficits, we crossed TRGL and AD model mice that develop either early-onset or late-onset disease pathology. In all five AD mouse models studied, we demonstrated early-appearing deficiencies of lysosomal vATPase activity, autophagy dysfunction in vulnerable neuron populations and accumulation of APP-βCTF and Aβ selectively within poorly acidified AL (pa-AL) well before extracellular β-amyloid deposition. Furthermore, we identified a unique autophagic stress response in more compromised neurons characterized by fulminant proliferation of AVs within perikarya and formation of large membrane blebs packed with Aβ/APP-βCTF-filled AVs. The strongly fluorescent petal-like blebs surrounding a DAPI-positive fluorescent nucleus generate flower-like profiles that we term ‘PANTHOS’ (poisonous flower). Notably, AV fusion with endoplasmic reticulum (ER) yields intraluminal formation of β-amyloid fibrils in a tubular network surrounding the nucleus, yielding morphologic features of a cored amyloid plaque within the intact neuron. Using an extensive array of imaging and histochemical techniques, we establish quantitatively that PANTHOS neurons are the origin of the vast majority of senile plaques in AD mouse models, thus prompting a reconsideration of the conventionally accepted sequence of events in plaque formation in AD.

## Results

### Detecting in vivo ALP dysfunction

**Fig. 1:**
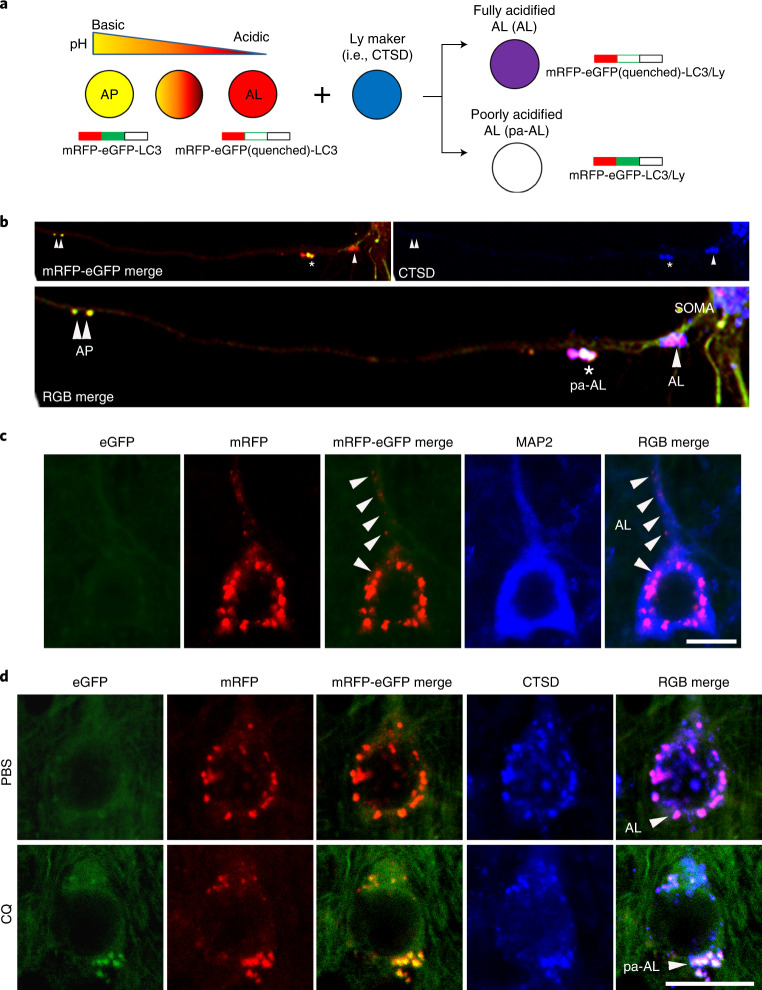
Design and expression of dual-tagged autophagy sensor in TRGL mouse brain. **a**, Schematic representation of the tfLC3 color change. The sensor is composed of pH-resistant mRFP, pH-sensitive eGFP and LC3. An acidic environment triggers the quenching of the eGFP signal, resulting in the conversion of net yellow signal to red-only signal. In combination with LY marker (pseudo-blue), fully acidified AL (AL) or poorly acidified AL (pa-AL) produce purple or white color, respectively. **b**, tfLC3 fluorescence change in primary neurons. APs (double arrowheads) were seen at distal levels of axons, and pa-ALs (asterisk) were seen at more proximal levels, whereas fully acidified ALs (arrowhead) were predominantly located near or in the perikaryon. **c**, Representative fluorescence images from neocortical layer V neurons of TRGL mice co-labeled with the cytoskeleton marker MAP2. Arrowhead denotes fully acidified AL (AL). Scale bar, 10 μm. **d**, Representative fluorescence images of the tfLC3 fluorescence change under lysosomal acidification altered conditions (CQ) in TRGL mouse brain. Arrowheads denote AL or pa-AL. Scale bar, 20 μm. **b**–**d**, Experiment was repeated three times independently with similar results.

A tandem mRFP-eGFP-LC3 transgene (tfLC3) driven by the THY-1 promoter is postnatally expressed specifically in neurons. tfLC3 is expressed approximately one-fold higher than endogenous LC3 levels and has no detectable effects on the ALP^13^. Like endogenous LC3, tfLC3 binds to AP membranes and persists after AP–LY fusion as an internalized substrate degraded within AL, ultimately yielding non-fluorescent LYs. The tfLC3 on AP fluoresces yellow-green (eGFP/mRFP) at the neutral pH of AP, but AL maturation upon fusion with LY^14^ acidifies the AL, causing fluorescence shifts from yellow to orange and then to red as eGFP fluorescence is quenched below pH 6.0 (ref. ^15^). LYs after autophagic clearance of fluorescent LC3 or after new LY biogenesis can be visualized by immunohistofluorescence (IHF) labeling with LY markers (for example, cathepsin D (CTSD) or LAMP 2) tagged with a third fluorophore. Notably, this third fluorophore also differentiates the yellow-fluorescing AP from an AP that fuses with an LY and is cathepsin-positive but fails to acidify adequately and, thus, fluoresces yellow by tfLC3 labeling alone (Fig. 1a)^13,14^. The latter profile is classified as a pa-AL.

AP maturation and acidification are most easily appreciated when the transition from AP to AL is protracted during retrograde axonal transport in primary neuronal cultures of TRGL mice (Fig. 1b). AVs are much fewer in vivo in the intact mature brain^16^. Fully acidified AL is concentrated within perikarya and proximal dendrites in neurons (Fig. 1c, arrowhead). ALs fluoresce purple (combined red and blue) in a three-fluorophore (RGB) analysis of neocortical perikarya, reflecting an efficient perikaryal acidification mechanism (Fig. 1d, top). To model an AL/LY acidification deficit in vivo and validate the tfLC3 probe in intact brain in vivo, 6-month-old TRGL mice were administered the amphiphilic weak base chloroquine (CQ) or the vehicle alone (controls) by intraventricular infusion for 5 days, and neurons in neocortical layers III–V were imaged (Fig. 1d). A rise in vesicle pH above 6.0 causes tfLC3-positive puncta to fluoresce yellow. Based on a green/red channel merge alone, these puncta would be mis-identified as AP; however, IHF with a CTSD antibody and Alexa Fluor 647 (pseudo-blue) secondary antibody identifies these puncta as CTSD-positive and, therefore, as pa-AL. In a three-channel merge, they fluoresce white (green, red and blue fluorescence) (Fig. 1d, RGB merge bottom), contrasting with the purple acidified AL in normal neurons (Fig. 1d, RGB merge top). LYs remain blue after CQ, reflecting their pH-insensitive detection by IHF (Fig. 1d). A computer algorithm^13^ determines for each vesicle the relative contributions of the three fluorophores based on their hue angle and saturation, which is a more precise objective representation of ‘color’ (and vesicle identity) than achieved by visual perception.

### AL acidification deficiency arises before β-amyloid deposits

**Fig. 2:**
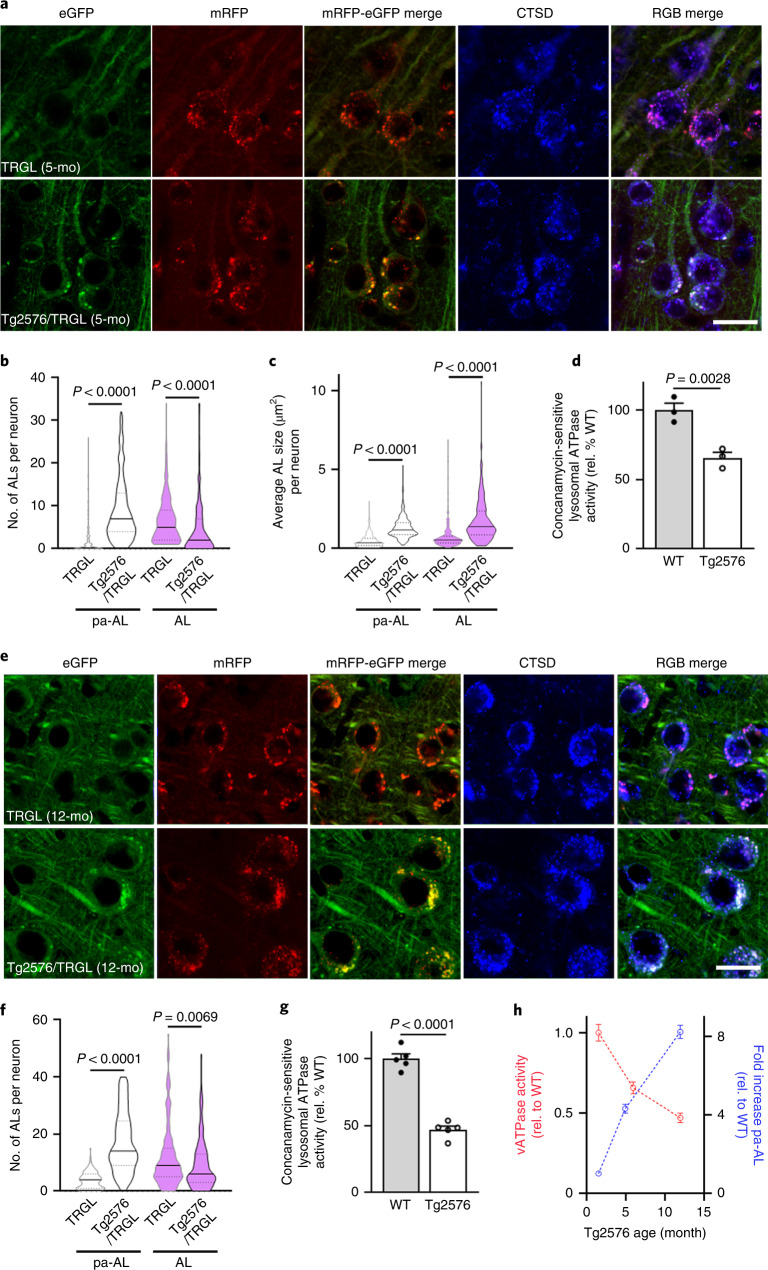
AL acidification deficits develop early in AD model mice and progress with age. **a**, Representative fluorescence images of tfLC3, co-labeled with CTSD, in neocortical neurons of 5-month-old TRGL and Tg2576/TRGL mouse brains. ALs exhibit a red or purple color without or with CTSD co-localization, respectively, whereas pa-ALs exhibit a yellow or white signal depending on CTSD co-label, respectively. Scale bar, 20 μm. **b**, Number of pa-ALs in 5-month-old Tg2576/TRGL is elevated compared to neurons in TRGL littermates. *n* = 243 (TRGL) and *n* = 245 (Tg2576/TRGL) neurons from three mice. **c**, pa-AL size in 5-month-old Tg2576/TRGL are larger than neurons in TRGL littermates. *n* = 243 (TRGL) and n = 245 (Tg2576/TRGL) neurons from three mice. **d**, Lysosomal vATPase activity is decreased in 6-month-old male Tg2576 compared to WT littermate neocortex. *n* = 3 mice. **e**, Representative fluorescence images of 12-month-old TRGL and Tg2576/TRGL mouse brains. Scale bar, 20 μm. **f**, Number of pa-ALs in 12-month-old Tg2576/TRGL are elevated compared to TRGL littermate neocortical neurons and to 5-month-old Tg2576/TRGL. *n* = 202 (TRGL) and n = 213 (Tg2576/TRGL) neurons from three mice. **g**, Lysosomal vATPase activity is decreased in 12-month-old male Tg2576 compared to WT littermates (and greater than in 6-month-old Tg2576). *n* = 5 mice. Violin plot colors correspond to the colors of the puncta (white: pa-AL; purple: AL). **h**, Time course analysis of vATPase activity and pa-AL number in Tg2576 mice. vATPase activity: *n* = 3 (1.6 months and 5 months) and *n* = 5 (12 months). pa-AL: *n* = 243 (1.6 months), *n* = 245 (5 months) and *n* = 213 (12 months). Quantitative data are presented as means ± s.e.m., unpaired *t*-test, two-tailed *P* value as indicated. **a**, **e**, Experiment was repeated three times independently with similar results. See also Extended Data Fig. 1. mo, month; rel., relative. Source data

**Extended Data Fig. 1:**
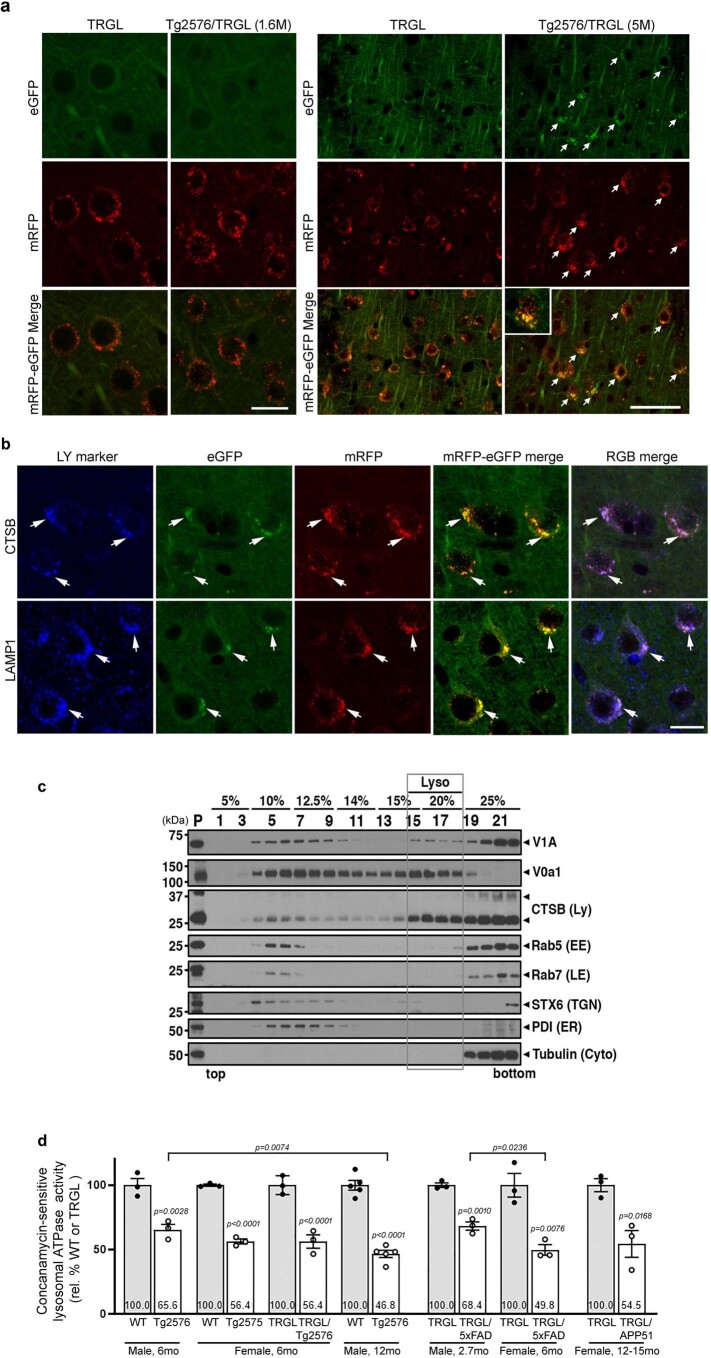
Early emergence of autolysosomal acidification deficits in Tg2576/TRGL mice brain. **a**. Representative fluorescence images from neocortical layer V neurons of TRGL and Tg2576/TRGL mice at two different ages. Neuronal perikarya of 1.6-month-old mice appeared normal in both genotypes, but yellow tfLC3 puncta accumulated in the perikarya of Tg2576/TRGL by 5 months of age (arrows). Scale bar 20 μm (left) or 50 μm (right). Experiment was repeated 3 times independently with similar results. **b**. Representative fluorescence images of tfLC3, co-labeled with CTSB or LAMP1, in neocortical neurons of 5-month-old TRGL and Tg2576/TRGL mouse brains. pa-AL exhibit a white signal depending on lysosome markers co-label (arrow). Scale bar 20 μm. **c**. Lysosome enriched fractions for lysosomal vATPase activity assay were isolated using a 25% OptiPrep gradient. Lysosome enriched fractions (grey box; #15~#18) were validated with various organelle markers. Experiment was repeated 3 times independently with similar results. **d**. Lysosomal vATPase activity of Tg2576/TRGL, 5xFAD/TRGL, and APP51/TRGL mouse cortex compared with littermate control neocortex. WT, M 6 mo (100±5.3 %), Tg2576, M 6mo (65.6±4.1 %), WT, F 6mo (100±0.8 %), Tg2576, F 6mo (56.4±1.9 %), TRGL, F 6mo (100±4.2 %), Tg2576/TRGL, F 6mo (56.4±5.2 %), WT, M 12mo (100±3.7 %), Tg2576, M 12mo (46.8±2.8 %), TRGL, M 2.7mo (100±1.8 %), 5xFAD/TRGL, M 2.7mo (68.4±3.2 %), TRGL, F 6mo (100±9.2 %), 5xFAD/TRGL, F 6mo (49.8±4.1 %), TRGL, F 12-15mo (100±5.0 %), APP51/TRGL, F 12-15mo (54.5±10.3 %). Number denotes mean value. n=3-5 mice. Quantitative data are presented as means ±S.E.M. unpaired t-test, two-tailed P value as indicated. Source data

We crossed TRGL mice^13^ with Tg2576 mice^17^, an AD model that develops β-amyloid plaques starting at 10~12 months of age. ALP patterns in 1.6-month-old Tg2576/TRGL crosses were indistinguishable from single-TRGL littermates (Extended Data Fig. 1a); however, by 5 months of age, more than 90% of neocortical layer III–V perikarya had acquired yellow fluorescent AVs in addition to acidified ALs (Extended Data Fig. 1a). CTSD co-labeling revealed that the yellow AVs are exclusively CTSD-positive and, therefore, pa-ALs (Fig. 2a, bottom panels). pa-AL was also positive for CTSB and the lysosomal membrane protein LAMP1 (Extended Data Fig. 1b). Hue-angle-based assignment and quantification of AV subtypes in neocortex^13^ revealed four-fold more pa-ALs in Tg2576/TRGL (9.0 ± 0.5 per neuronal cross-section) than in TRGL (2.1 ± 0.3), significantly fewer mature ALs (4.4 ± 0.4 versus 6.6 ± 0.3 per neuronal cross-section) (Fig. 2b) and increased size of pa-ALs and ALs (1.3 ± 0.04 versus 0.48 ± 0.03 and 1.75 ± 0.09 versus 0.74 ± 0.05, respectively) (Fig. 2c). By 12 months, perikaryal pa-ALs further increased in Tg2576/TRGL (17.2 ± 0.7 per neuronal cross-section) (Fig. 2e,f). To further document AL acidification deficits in Tg2576 brain, we isolated AL/LY-enriched fractions by OptiPrep density centrifugation (Extended Data Fig. 1c) and assayed their ATPase activity^18^. Consistent with observed pH deficits, vATPase activity in LY/AL of 6-month-old Tg2576 was decreased (65.6 ± 4.1%) compared to that of age-matched wild-type (WT) littermates (Fig. 2d) and decreased further by 12 months in Tg2576 mouse brain (45.3 ± 3.7% relative to WT) (Fig. 2g). ATPase activity was similarly reduced in brains from two other mouse models of AD (5xFAD and APP51) (Extended Data Fig. 1d). The time course graph indicates age-dependent increased prevalence of pa-AL while vATPase activity declines (Fig. 2h).

### APP-βCTF/Aβ accumulate in pa-AL at early stages of disease

**Fig. 3:**
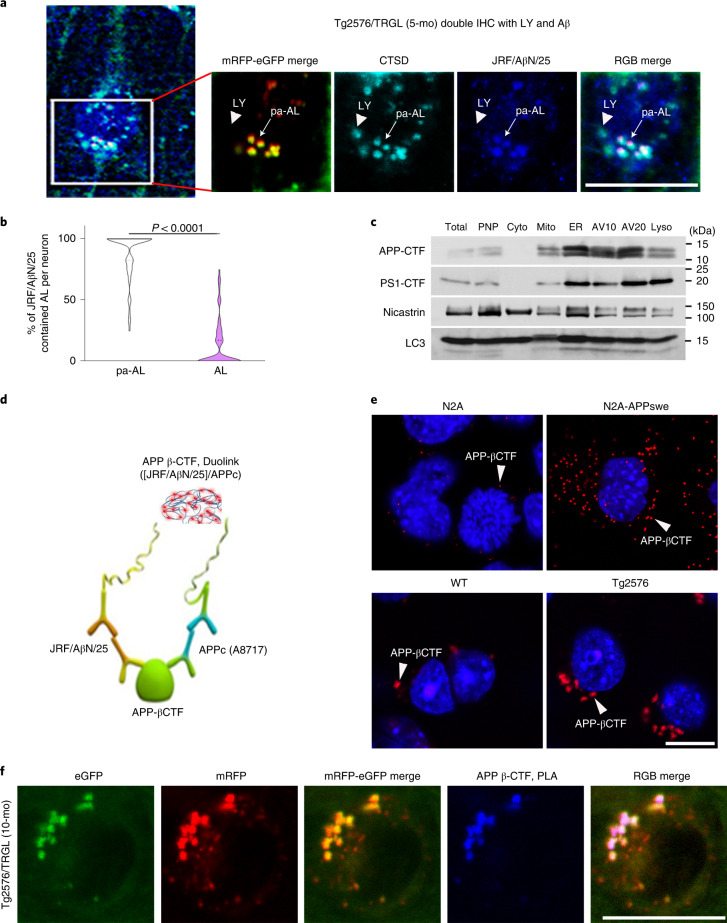
Intraneuronal APP-βCTF/Aβ accumulates selectively within pa-AL in AD mice. **a**, Immunofluorescence co-labeling of 5-month-old Tg2576/TRGL mouse brain neurons with a CTSD antibody and JRF/AβN/25 antibody against APP-βCTF/Aβ. APP-βCTF/Aβ accumulates in enlarged pa-ALs producing a white signal (arrow), whereas it is absent from LYs (arrowhead). Scale bar, 20 μm. **b**, Percentage of AL and pa-AL subtypes positive for JRF/AβN/25 immunoreactivity in neurons of 5-month-old Tg2576/TRGL mouse brains. *n* = 66 neurons from three mice. Violin plot colors correspond to the colors of the puncta (white: pa-AL; purple: AL). Quantitative data are presented as means ± s.e.m., unpaired *t*-test, two-tailed *P* value as indicated. **c**, AV fractionation from 10-month-old Tg2576 mice. Fractions were obtained by pooling five mouse brains. The experiment was repeated two times independently with similar results. **d**, Schematic representation of the PLA performed using JRF/AβN/25 for APP-βCTF N-terminus and APPc for APP-βCTF C-terminus. **e**, Representative PLA fluorescence images from N2A-APPswe cells and 10-month-old Tg2576 mouse brain compared to WT controls. Arrowheads denote PLA signal for APP-βCTF. Scale bar, 20 μm. **f**, Representative PLA fluorescence images from Tg2576/TRGL mouse brain. PLA signals were co-localized with pa-AL, resulting in white puncta. Scale bar, 20 μm. **a**, **c**, **e**, **f**, The experiment was repeated three times independently with similar results. See also Extended Data Fig. 2. IHC, immunohistochemistry; mo, month. Source data

**Extended Data Fig. 2:**
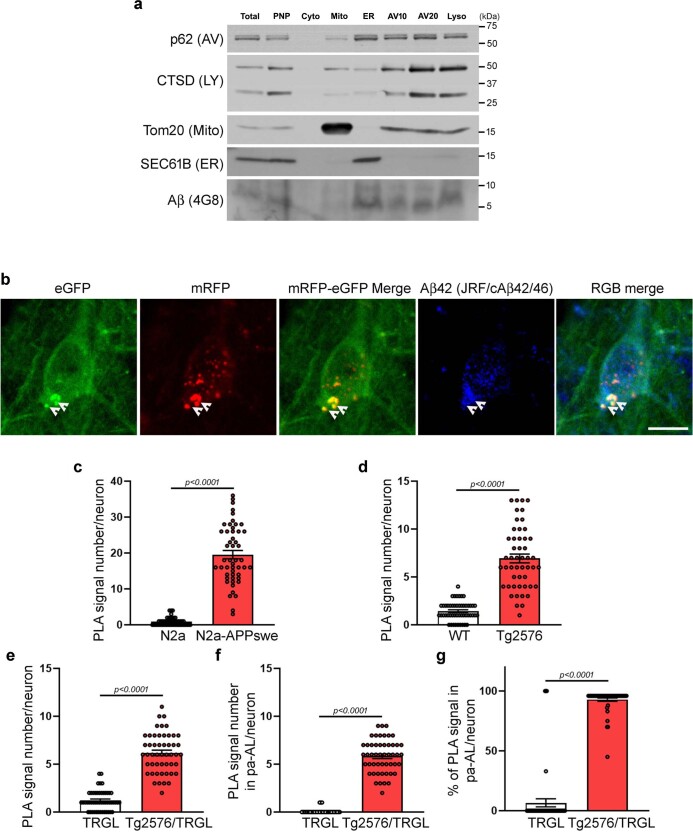
Intraneuronal APP-βCTF/Aβ accumulates selectively within pa-AL in AD mice. **a**. AV fractionation from 10-month-old Tg2576 mice. Fractions were validated by organelle markers (lysosome: CTSD, mitochondria: Tom20, ER: SEC61B, AV: p62) and anti-Aβ antibody 4G8. Experiment was repeated 2 times independently with similar results. **b**. Immunofluorescence co-labeling of 5-month-old Tg2576/TRGL mouse brain neurons with an antibody against Aβ1−42 (JRF/cAβ42/26). Aβ accumulates in enlarged pa-AL producing a white signal (arrowhead). Experiment was repeated 3 times independently with similar results. Scale bar 20 μm. (**c**) Quantitation graph of the PLA fluorescence per neuron from N2A-APPswe cell (N2a (0.9±0.2), N2a APPswe (19.6±1.1)) and (**d**) 10-month-old Tg2576 mouse brain compared with WT controls. WT (1.4±0.1), Tg2576 (6.9±0.5). n=50 cells per each. **e-g**, Quantitation graph of the PLA fluorescence per neuron from 10-month-old Tg2576/TRGL. **e**. Total number of PLA signal per neuron. TRGL (1.2±0.1), Tg2576/TRGL (6.2±0.3). **(f)** Number of the PLA signal in pa-AL per neuron. TRGL-pa-AL (0.1±0.0), Tg2576/TRGL-pa-AL (5.8±0.2). (**g**) Percentage of PLA signal in pa-AL in neuron compared with WT controls. TRGL-pa-AL (6.7±3.4 %), Tg2576/TRGL-pa-AL (92.9±1.3 %). n=50 cells. Quantitative data are presented as means ±S.E.M. unpaired t-test, two-tailed P value as indicated. **a-d**: Experiment was repeated 3 times independently with similar results. Source data

APP-βCTF and Aβ accumulate intracellularly before β-amyloid is deposited extracellularly in AD, with the endosomal–lysosomal system representing the main subcellular site for their generation^19–21^. To relate APP-βCTF/Aβ intracellular accumulation to early AL acidification deficits in Tg2576 mice, we localized APP metabolites within AV subtypes using a monoclonal antibody (JRF/AβN/25) that detects APP-βCTF and Aβ^22^. By 5 months, 40% of layer III–V neocortical perikarya in Tg2576/TRGL mice contained Aβ/APP-βCTF-positive puncta (Fig. 3a), which were almost exclusively pa-AL (88.6 ± 2.4%) based on CTSD co-immunolabeling and imaging of four fluorophores (Fig. 3a, arrows, and Fig. 3b). Immunoblot analyses on subcellular fractions from Tg2576 brains confirmed that LC3-II enriched AV fractions contain abundant APP-βCTF as well as γ-secretase components (presenilin 1 and nicastrin) (Fig. 3c) and Aβ (Extended Data Fig. 2a). Aβ localization in AVs was further validated by Aβ1-42 antibody (JRF/cAβ42/26) (Extended Data Fig. 2b, arrowhead). Also, APP-βCTF localization in AVs was further validated by an in situ proximity ligation assay (PLA) using a modified Duolink technology (Methods) involving two primary antibodies directed against different epitopes (N-terminus or C-terminus) on APP-βCTF (Fig. 3d). PLA fluorescence (red) detected APP-βCTF in APPswe-overexpressing N2A cells and Tg2576 neurons at considerably higher levels than in controls (Fig. 3e, arrowheads, and Extended Data Fig. 2c,d). Notably, PLA signal (blue) revealed that APP-βCTF selectively accumulated in ALs that were poorly acidified in Tg2576/TRGL perikarya (92.9 ± 1.3%, *n* = 50 neurons) (Fig. 3f and Extended Data Fig. 2e–g).

### Progressively compromised neurons massively accumulate pa-AL

**Fig. 4:**
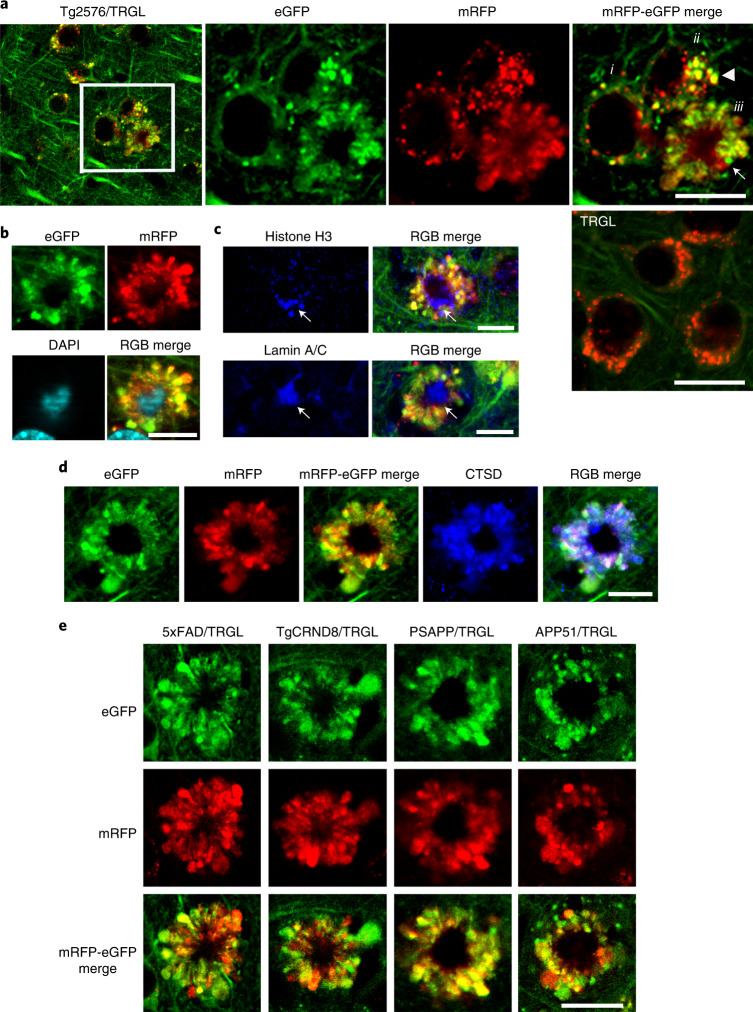
tfLC3 probe reveals a unique pattern of autophagic stress, AL pH deficit and plasma membrane blebbing (‘PANTHOS’) in five different AD mouse models. **a**, Representative tfLC3 fluorescence images of 10-month-old Tg2576/TRGL mouse brain depicting neurons at three stages of PANTHOS (i: early pH change in AL; ii: focal PM bulging as pa-ALs enlarge and proliferate (arrowhead); iii: full PANTHOS pattern (arrow)). (See graphic representation of these stages in Extended Data Fig. 8). A control TRGL neuron (5th panel in **a**) exhibits fully acidified ALs. Scale bar, 20 μm. **b**, Staining of PANTHOS neurons using nuclear marker (DAPI) in 10-month-old Tg2576/TRGL mouse brain. Scale bar, 10 μm. **c**, IHF staining of PANTHOS neurons using nuclear markers (histone H3 and lamin A/C) in 10-month-old Tg2576/TRGL mouse brain. Scale bar, 10 μm. See also Extended Data Fig. 2. **d**, IHF staining of LY marker (CTSD) in 10-month-old Tg2576/TRGL mouse brain. Scale bar, 10 μm. **e**, PANTHOS pattern is conserved across four additional AD mouse models. Male 5xFAD/TRGL (2.7 months) and male TgCRND8/TRGL (1.9 months) and female PSAPP/TRGL (3.1 months) and female APP51/TRGL (20 months) were imaged. Scale bar, 10 μm. **a**–**e**, The experiment was repeated three times independently with similar results. See also Extended Data Fig. 3. PM, plasma membrane.

**Extended Data Fig. 3:**
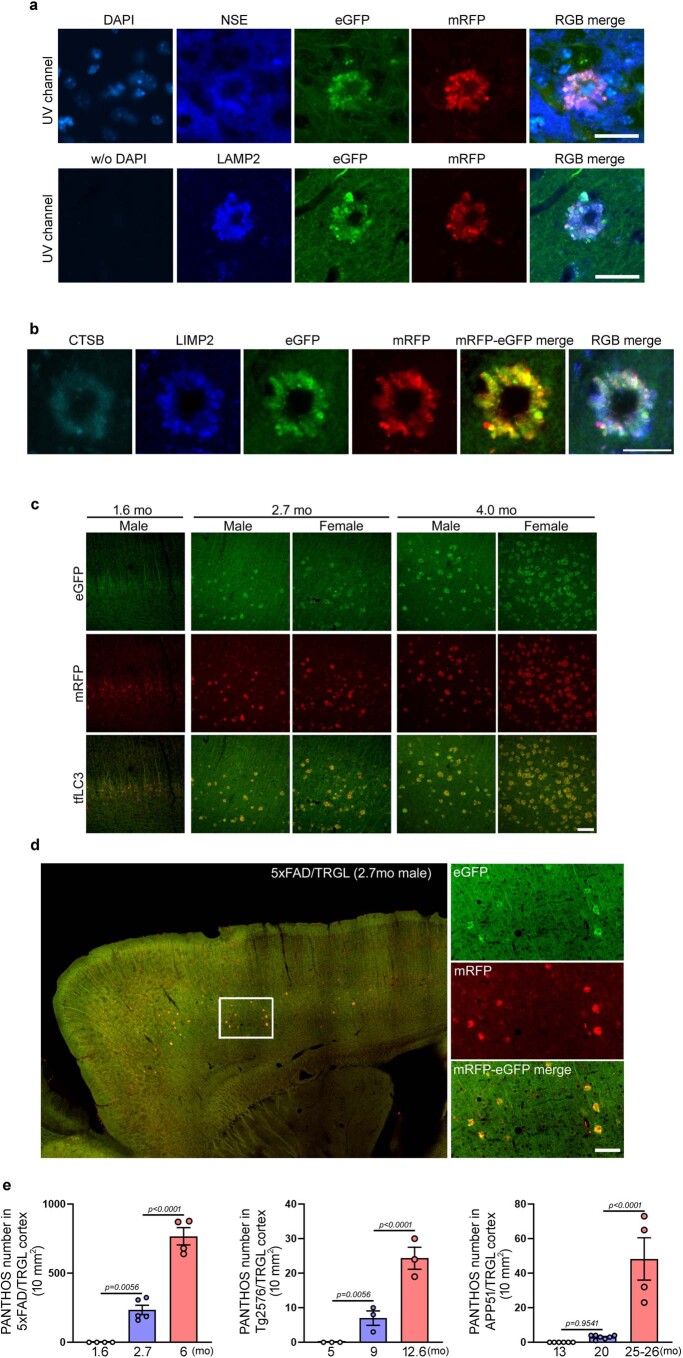
Neuron-specific origin of PANTHOS and age/sex dependent PANTHOS neuron proliferation in brains of 5xFAD/TRGL mice. **a**. PANTHOS neurons were immunolabeled with neuron specific enolase (NSE) which detects neuronal populations, especially cell bodies, and were counter-stained with DAPI. NSE IHC indicates that PANTHOS neurons were NSE-positive. The UV channel did not produce any autofluorescence from PANTHOS neurons when DAPI counterstaining was not done in 2.7-month-old 5xFAD/TRGL mouse brain. Scale bar 20 μm. **b**. PANTHOS neurons were immunolabeled with lysosome marker CTSB and LIMP2 in 2.7-month-old 5xFAD/TRGL mouse brain. Scale bar 20 μm. **c**. tfLC3 signal in cerebral cortex at three ages in 5xFAD/TRGL male and female mice demonstrating age- and sex- dependent proliferation of PANTHOS neurons. **d**. Overview of PANTHOS neuron distribution in cerebral cortex of the 2.7-month-old 5xFAD/TRGL male mouse (top panel) and dual channel higher magnification (bottom). Scale bar 50 μm. **e**. Age dependent increased prevalence of the PANTHOS profiles in various AD mouse models. 5xFAD/TRGL: 1.6 mo (0±0), 2.7 mo (2340±33.8), 6 mo (767±62.3); Tg2576/TRGL: 5 mo (0±0), 9 mo (7.0±2.1), 12.6 mo (24.3±3.2); APP51/TRGL: 13 mo (0±0), 20 mo (3.0±0.4), 25-26 mo (48.3±12.2). Data points indicate mouse numbers; mo. denotes age in months. Quantitative data are presented as means ±S.E.M. unpaired t-test, two-tailed P value as indicated. **a-d**: Experiment was repeated 3 times independently with similar results. Source data

In 10-month-old Tg2576/TRGL mice, a subpopulation of neocortical neurons (layer III–V) began to accumulate substantially enlarged pa-ALs, which bulge the plasma membrane outward (Fig. 4a, enlarged right panel, arrowhead). The further massive proliferation of LC3-positive vesicles was accompanied by formation of large strongly fluorescent membrane blebs that project from the plasma membrane and expand perikaryal circumference. A central nuclear region devoid of LC3 fluorescence (Fig. 4a) could be labeled by nuclear markers, including DAPI, histone H3 or lamin A/C (Fig. 4b,c). The absence of autofluorescence in this nuclear area excluded the possibility that DAPI signal was non-specific autofluorescence due to amyloid (Extended Data Fig. 3a). Most AVs in affected perikarya were LY-marker-positive by IHF, indicating that they were pa-ALs (Fig. 4d and Extended Data 3b), which reflects a severe deficit of AL maturation and acidification.

**Fig. 5:**
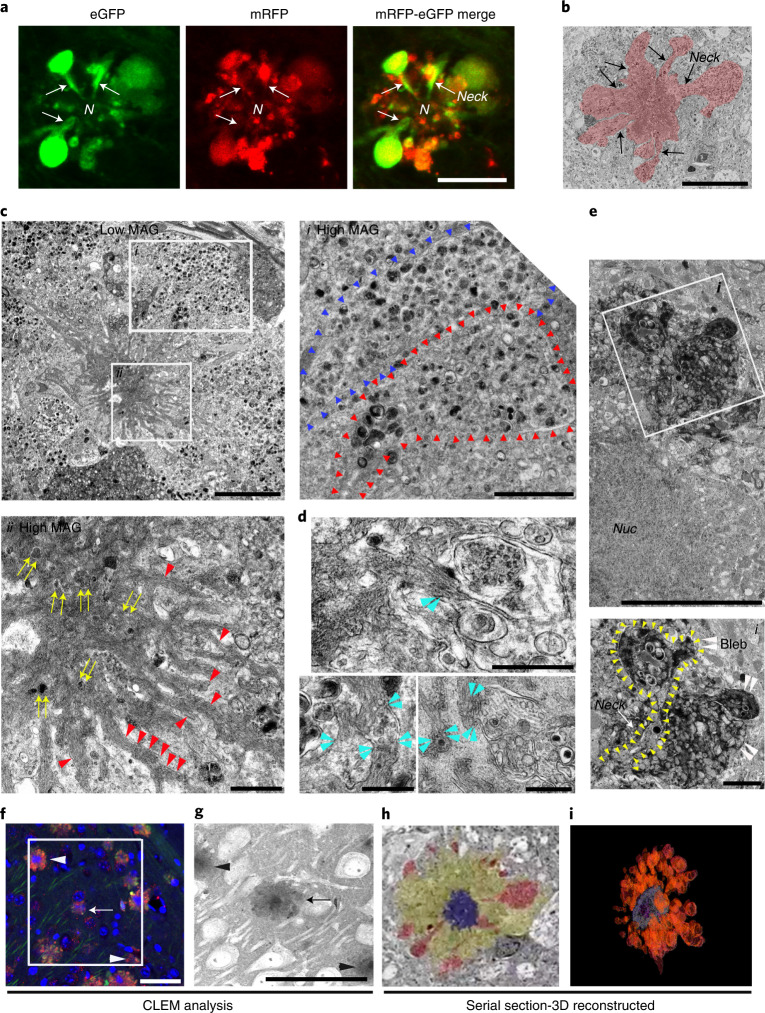
Ultrastructural characterization of PANTHOS neurons in an AD mouse model. Confocal image of a PANTHOS neuron exhibiting many tfLC3-positive (AV-filled) blebs with tapered necks arising from the perikaryon. N denotes nucleus area. See also in Extended Data Fig. 4. Scale bar, 10 μm. **b**, Representative EM image of a PANTHOS neuron depicting AV-filled blebs projecting from the perikaryal plasma membrane via necks that are continuous with perikaryal cytoplasm (arrow). 2.7-month-old 5xFAD/TRGL mouse brain. Scale bar, 20 μm. **c**, EM image of a PANTHOS neuron from a 5-month-old 5xFAD/TRGL mouse brain. Scale bar, 5 μm. Box i: AV-filled peripheral plasmalemmal blebs (blebs membrane boundary: arrowheads). Scale bar, 2 μm. Box ii: a centrally located electron-dense network of radiating membrane-bound tubular extensions (red arrowheads) containing incorporated AVs (yellow arrows). Scale bar, 1 μm. **d**, EM images for the spatial relationship between AVs and tubular extensions within which thin fiber bundles are visible (light blue arrowheads: AV/tubule contact sites). Scale bar, 500 nm. Full-resolution images for **c** and **d** are presented as Extended Data Fig. 5. **e**, Representative perikaryal blebs extending from the plasma membrane of a PANTHOS neuron. PS/APP mouse brain, labeling by acid phosphatase (ACPase) cytochemistry, a marker of AL/LY, reveals the fulminant autophagy pathology (mainly ALs) segregated into blebs. Scale bar, 5 μm. Box i: Enlarged EM image of the ROI area (box) depicting a bleb (white arrowhead) and long cytoplasmic neck (outlined by yellow arrowheads). Scale bar, 1 μm. **f**, Immunohistochemistry image of the ROI (box) used for serial SEM imaging of the 2.7-month-old 5xFAD/TRGL mouse brain. Scale bar, 40 μm. **g**, *z*-stacked serial SEM image, 370–430, of the ROI area. Scale bar, 40 μm. Arrow indicates the PANTHOS of interest; arrowheads indicate adjusted reference PANTHOS. Bleb tracing (**h**) and 3D reconstruction of the PANTHOS (**i**) using IMOD modeling. The experiment was repeated three (**a**–**e**) or two (**f**–**i**) times independently with similar results. See also Supplementary Fig. 1 and Video 1.

We observed an identical autophagic neurodegenerative pattern in five different mouse models of AD, including models with accelerated neuropathology onset (5xFAD, TgCRND8 and PSAPP) or delayed onset (Tg2576 and APP51—an exceptionally late-onset model expressing hAPPwt)^23^ (Fig. 4e). 5xFAD/TRGL mice develop robust ALP disruption and neuronal degeneration at an early age (starting after 2 months depending on sex)^24–26^ (Extended Data Fig. 3c–e) and in a more reproducible pattern than in Tg2576 or APP51 mice (Extended Data Fig. 3e). We used this model in further investigations on the relationship between the development of LC3-positive membrane blebs and disease progression, including quantitative amyloid plaque pathology. To our knowledge, similar huge AV-filled perikaryal membrane protrusions, as further defined ultrastructurally (Fig. 5), have not been previously described in a neurodegenerative state^27^. Because these rosettes of large fluorescent blebs surrounding a central DAPI-positive nucleus resemble petals of a flower, we have termed this unique degenerative process PANTHOS and refer to the affected cells as PANTHOS neurons.

### PANTHOS—a unique pattern of neurodegeneration in AD

**Fig. 6:**
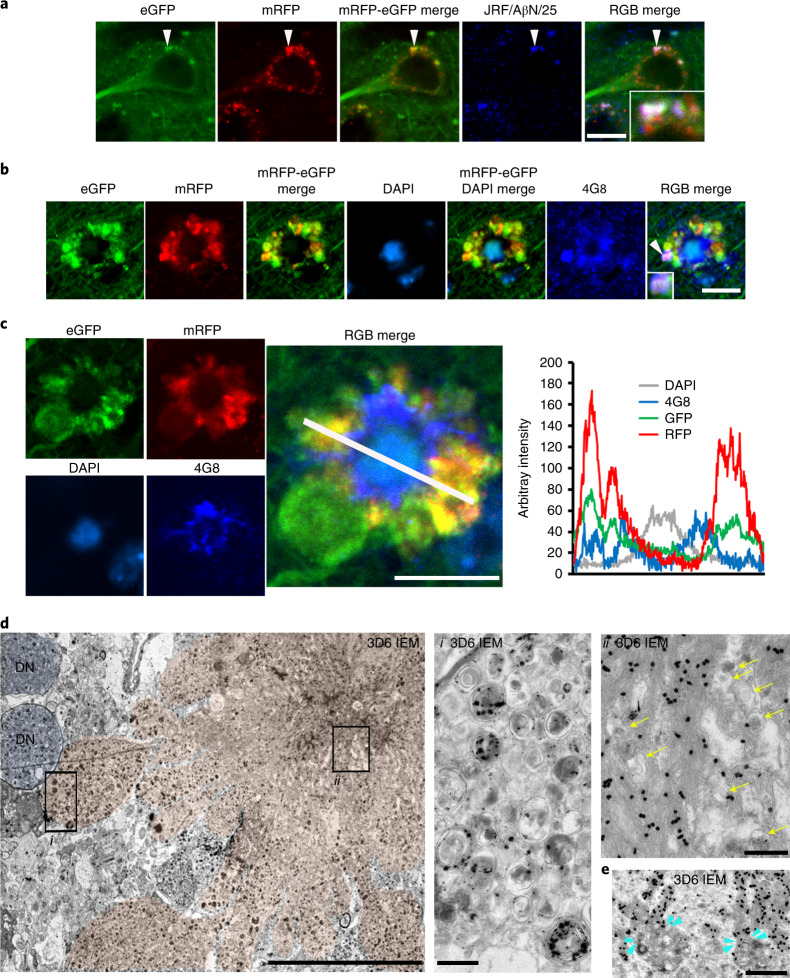
Evolution of intraneuronal β-amyloid accretion and distribution in PANTHOS neurons in brains of AD mouse models. **a**, IHF co-labeling of 2.7-month-old male 5xFAD/TRGL mouse brain neurons with JRF/AβN/25 monoclonal antibody against APP-βCTF/Aβ. Scale bar, 10 μm. **b**, IHF labeling of Aβ (4G8) and DAPI stain. Perinuclear intraneuronal Aβ accumulation surrounding a visible DAPI-positive nucleus within a PANTHOS neuron. Inset depicts Aβ in a bleb of the PANTHOS neuron. Scale bar, 10 μm. **c**, Immunofluorescence staining of a DAPI-labeled PANTHOS neuron using 4G8 antibody followed by fluorescence intensity analysis. Perinuclear Aβ accumulates within a PANTHOS neuron. The white line in the merged image indicates the scan path through the PANTHOS neuron from which fluorescence intensity is determined spatially for each fluorophore. Scale bar, 10 μm. **d**, Representative Aβ IEM (3D6) image demonstrates extensive AV-filled blebbing of the PM in a PANTHOS neuron (colorized light pink) and, by comparison, two profiles (blue coloration) tentatively identified as DNs in a 5-month-old 5xFAD/TRGL mouse brain. Scale bar, 10 μm. Box i depicts Aβ immunoreactive AVs in the bleb. Box ii depicts overlap of Aβ immunoreactivity with the central nuclear area that also displays the electron-dense network of radiating membrane-bound tubular extensions, which are strongly Aβ immunoreactive. Yellow arrows indicate AVs incorporated into the central amyloid-positive network. Scale bar, 500 nm. **e**, Representative amyloid (3D6) IEM image. Light-blue arrowheads denote vesicle and amyloid bundle contact sites. **a**–**d**, The experiment was repeated three times independently with similar results. Scale bar, 1 μm. See also Extended Data Fig. 6. PM, plasma membrane.

**Extended Data Fig. 4:**
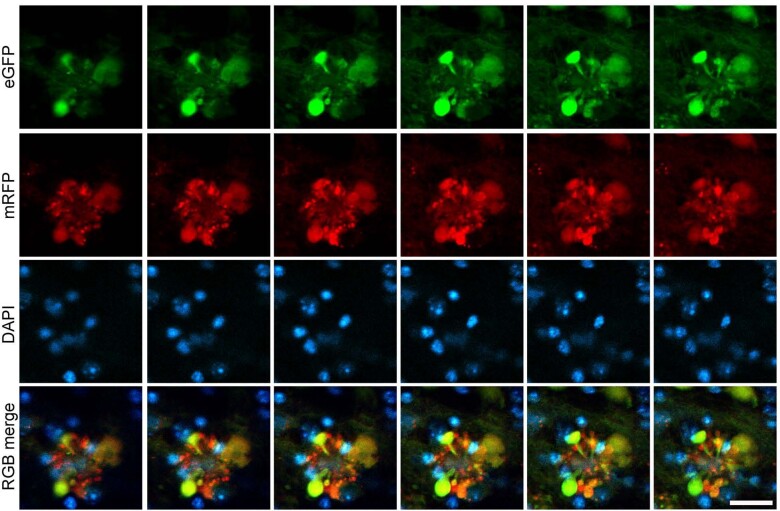
Tomographic rendering of the PANTHOS neuron from a 5xFAD/TRGL mouse brain shown in Fig. 5a. Serial z-stacked image (1 μm thick, number *z1*–*z6*) showing a flower shape structure of a PANTHOS neuron displaying the strongly fluorescent blebs with tapered necks arising from the perikaryal plasma membrane. The neuron is from the cerebral cortex layer V of 2.7-month-old male 5xFAD/TRGL mice. Scale bar 10 μm. Experiment was repeated 3 times independently with similar results.

**Extended Data Fig. 5:**
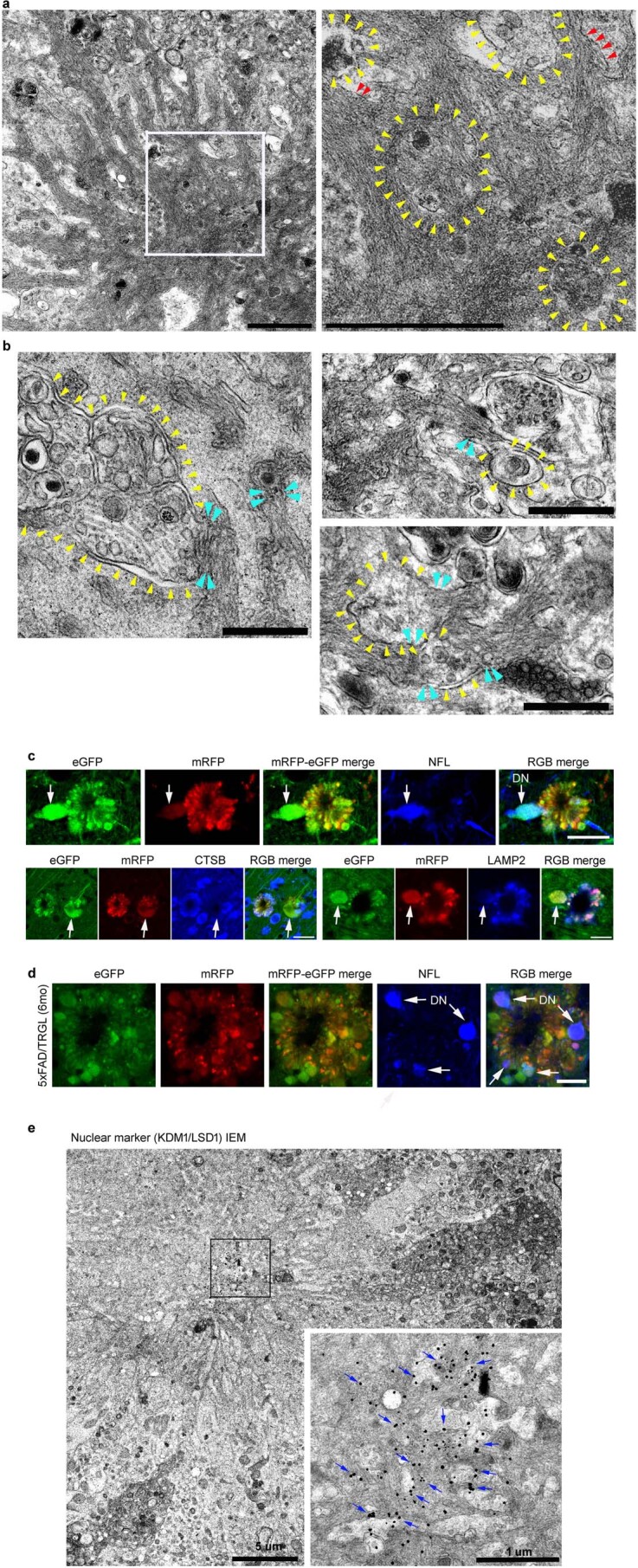
High resolution EM images of Fig. 5 panels reveal the contribution of AL fusion with a tubular central perinuclear network of strong Aβ/APP-βCTF IR. **a**. Enlarged EM image of Fig. 5c-*ii*. AVs indicated with yellow arrowheads in continuity with a membranous tubular network containing fibrous bundles (red arrowheads). Scale bar 1 μm. **b**. Full resolution image of Fig. 5d revealing continuity of AVs (yellow arrowheads) and the tubular network (light-blue arrowheads) in greater detail. Scale bar 500 nm. **c**. IHF labeling with antibodies to the neuronal cytoskeleton protein NFL and lysosomes (CTSB, LAMP2) in 2.7-month-old 5xFAD/TRGL mouse brain. NFL positive swollen process projecting peripherally from the PANTHOS neuron contrasts with the perikaryal blebs which have undetectable NFL signal consistent with the NFL process being a dystrophic axon (arrow). Scale bar 20 μm. **d**. IHF labeling with neuronal cytoskeleton protein NFL in 6-month-old 5xFAD/TRGL mouse brain. NFL positive swollen DN-like profiles are characteristically located at the periphery of the PANTHOS neuron. Scale bar 20 μm. **e**. IEM detection of strong immunolabeling for the nuclear marker (KDMA/LSD1 - blue arrows in box inset) in the area of a nucleus no longer identifiable morphologically in a PANTHOS neuron. Scale bar 5 μm and 1 μm (enlarged ROI). **a-e**: Experiment was repeated 3 times independently with similar results.

The greater resolution of autophagic profiles afforded by the tfLC3 probe allowed us to visualize by confocal imaging the AV-filled blebs extending directly from the perikaryal cytoplasm of PANTHOS neurons via necks that taper toward the center of the perikaryon (Fig. 5a and Extended Data Fig. 4). Electron microscopy (EM) analysis on brains of 5xFAD/TRGL mice confirmed the continuity of blebs with the perikaryal cytoplasm and identified AVs as the principal constituents within blebs (Fig. 5b). Perikaryal blebs exhibit long membrane-bound necks extending from the soma of the PANTHOS neuron (Fig. 5c, box i, outline with arrowheads). Additional features of PANTHOS neurons at higher EM resolution include a centrally located electron-dense network of radiating membrane-bound tubular extensions containing partially fused and fully incorporated AVs (Fig. 5c, box ii: yellow arrows, and Extended Data Fig. 5a, inset: yellow arrowheads) as well as bundles of 6-nm fibers (Fig. 5c, box ii, and Extended Data Fig. 5a, red arrowheads) that are strongly Aβ immunoreactive (Fig. 6d, box ii). In other EM images, AVs and Aβ-positive fiber-containing tubular extensions are seen to be in the process of fusing (Fig. 5d and Extended Data Fig. 5b, light-blue arrowheads).

EM analysis of brain sections labeled histochemically for the lysosomal enzyme acid phosphatase (ACPase) further confirmed the identity of most AVs in blebs as strongly ACPase-positive ALs, including those within the tapered bleb necks connecting blebs to the cytoplasm of the degenerating perikaryon (Fig. 5e, inset: yellow arrowheads). Although the asymmetric morphology of perikaryal blebs and their evident cytoplasmic origin distinguish them from DNs, blebs were further distinguished from DNs, which are enriched with neurofilaments, exhibit weak signal for lysosomal markers (CTSD and LAMP2) as shown by IHF (Extended Data Fig. 5c, arrow) and are infrequent compared to perikaryal blebs around PANTHOS neurons (Extended Data Fig. 5d).

To further establish the perikaryal origin of the many AV-filled blebbing profiles, we performed correlative light electron microscopy (CLEM) together with serial block-face scanning EM imaging using an Apreo scanning electron microscope. Reconstruction of more than 500 *z*-plane images recreated the entire PANTHOS neuron in three dimensions (Supplementary Fig. 1 and Movie 1). A stacked EM image sequence from 370 to 430 of region of interest (ROI) area (Fig. 5f) confirmed that the sizes of early-stage PANTHOS profiles approximate the size of normal neurons (Fig. 5g), but these profiles have expanded circumference as perikaryal blebbing becomes more extensive (Fig. 5h). The DAPI-positive center area of PANTHOS neurons approximates the sizes of the electron-dense centrally located areas in the stacked EM image (Fig. 5g). ImmunoEM analyses with the nuclear marker KDM1/LSD1 confirmed the existence of nuclear remnants in the central area by detecting strong immunoreactivity in the same central area even after nuclear integrity was extensively disrupted (Extended Data Fig. 5e). A movie sequence through these serial sections clearly visualized dozens of AV-filled membrane blebs arising within the cytoplasm from tapered necks that expand into large bulbous projections (blebs) from the perikaryon (Supplementary Movie 1), as shown in a colorized section from the full set (Fig. 5h). A 3D reconstruction modeling illustrates the extensive blebbing of the perikaryon (Fig. 5i).

### PANTHOS neurons are the principal origin of amyloid plaques

**Extended Data Fig. 6:**
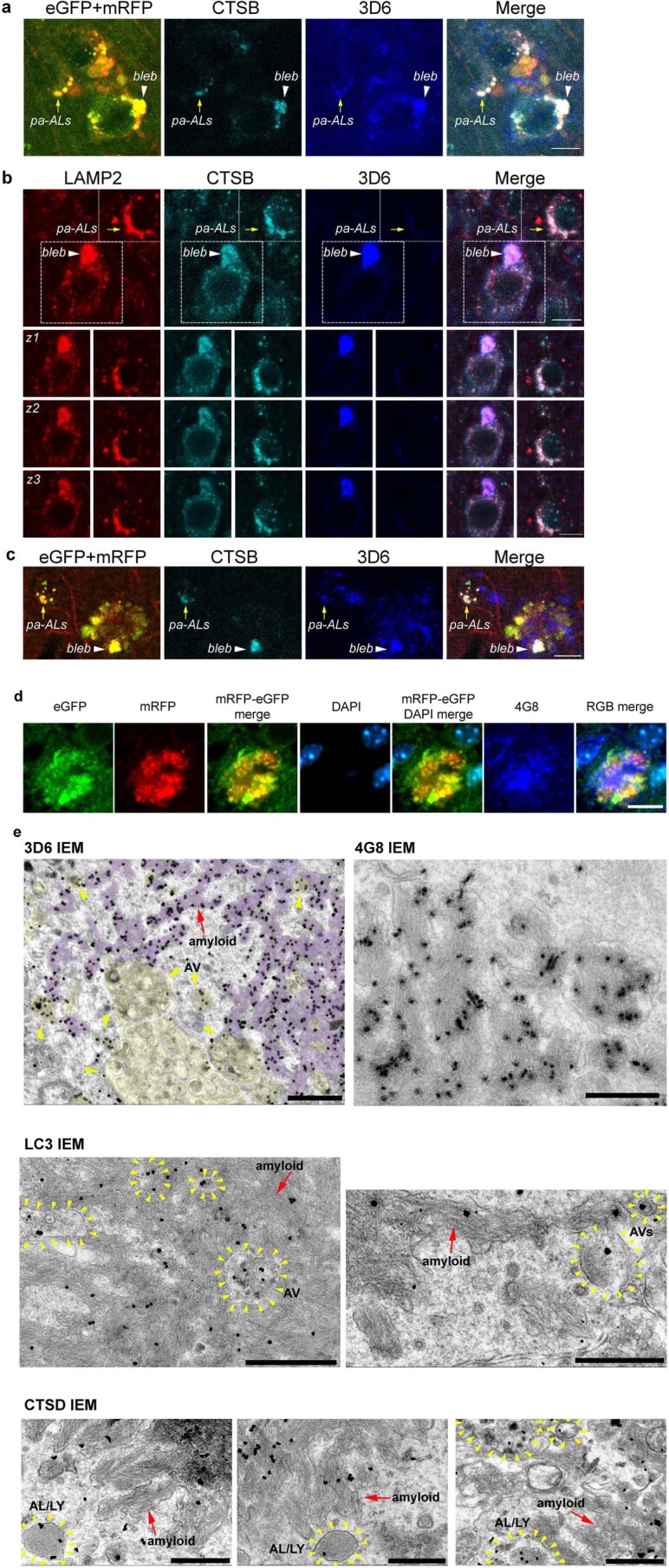
The progression of PANTHOS formation in relation to amyloid in AD mouse model brains and amyloid fiber network IEM characterization. **a**. IHF labeling of 28-month-old APP51/TRGL layer V cortical neurons with LY marker (CTSB) and 3D6 monoclonal antibody against APP-βCTF/Aβ. Representative plane from a Z-stack (*see* also Extended Data Fig. 7a) of an early stage: 3D6 accumulates in CTSB positive perikaryal pa-AL of a normal looking cell (*pa-AL*, yellow arrow) and in those of a bleb-forming cell (*bleb*, white arrowhead). Scale bar 10 μm. **b**. IHF labeling of 30-month-old APP51 layer V cortical neurons with LY membrane marker (LAMP2), CTSB and 3D6. Representative single plane (top panel) and respective Z-stack series (1 mm-thick *z1*-*z3*, 2^nd^ to 4^th^ panel) of an intermediate stage: 3D6 accumulates in a LAMP2 and CTSB double-positive bleb originated from the perikaryal protrusion of a degenerating neuron (arrowhead and respective series), as opposed to a 3D6-negative neuron with normal perikaryal morphology (arrow and respective series). Scale bar 10 μm. **c**. IHF labeling of 28-month-old APP51/TRGL layer V cortical neurons with CTSB and 3D6. Representative IHF image of late stage: 3D6 co-localizes with CTSB in a bleb containing pa-AL of a mature PANTHOS neuron (arrowhead), maintaining a similar spatial segregation of 3D6 and CTSB immunoreactivity as seen in earlier stages (*pa-AL*). Filamentous/fibrillar 3D6 signal also emanates from the center of the PANTHOS. Scale bar 10 μm. **d**. IHF labeling of APP-βCTF/Aβ (4G8) in 5-month-old 5xFAD/TRGL mouse brain. Representative IHF image of a late stage PANTHOS neuron with intraneuronal Aβ occupying the central area with a faded/disappeared nuclear-DAPI fluorescence. Scale bar 10 μm. **e**. Representative amyloid (3D6) (that is, full view images for the one shown in Fig. 6f), amyloid (4G8), AV (LC3), and AL/LY (CTSD) IEM images of 5-month-old 5xFAD/TRGL mouse brain. Yellow arrowheads denote AVs and red arrows denote amyloid bundles. Scale bar 1 μm (3D6, CTSD) and 500 nm (LC3). **a-e**: Experiment was repeated 3 times independently with similar results.

In 5xFAD/TRGL mice, Aβ and APP-βCTF accumulate selectively within pa-ALs before β-amyloid plaques appear (Fig. 6a, arrowheads), as in Tg2576 mice (Fig. 3). Transition of neurons to a PANTHOS pattern is accompanied by robust accretion of perinuclear Aβ/APP-βCTF immunoreactivity. Co-labeling of these PANTHOS neurons with DAPI and anti-β-amyloid antibody (4G8) identified a 4G8-positive corona surrounding a DAPI-positive nucleus remnant at the center of most affected perikarya (Fig. 6b). The progression of PANTHOS formation with respect to β-amyloid accretion was further confirmed in the late-onset AD mouse model APP51 (Extended Data Fig. 6a–c).

In 5xFAD/TRGL mice, quantitative spectral analysis of the PANTHOS neuron’s central area discriminated DAPI fluorescence from fluorescence due to 4G8 immunolabeling (Fig. 6c). At more advanced stages of PANTHOS, DAPI fluorescence gradually disappears as more β-amyloid accumulates centrally (Extended Data Fig. 6d). Ultrastructural and 3D6 immunoelectron microscopy (IEM) analyses localized this central accretion of Aβ immunoreactivity (Fig. 6d) within intraneuronal membranous tubular profiles (Fig. 6d, box i). Within many of these same profiles, 3D6-positive bundles of fibrils, with widths of around 10 nm, approximated the known diameters of fibrillar β-amyloid^28^ (Fig. 6d, box ii). Resembling the PANTHOS morphologies in Fig. 5b, Aβ IEM of a PANTHOS neuron with 3D6 additionally detected 3D6-positive AVs packed into perikaryal blebs. Perikaryal AVs were also shown to be continuous with, and incorporated into, the central Aβ-positive network of membrane tubular structures (Fig. 6e and Extended Data Fig. 6e (3D6 and 4G8 IEM)). IEM with antibodies to either LC3 or CTSD confirmed that these vacuoles are AVs (Extended Data Fig. 6e). ER, a key source for AP membrane components, is increasingly mobilized to supply membrane for new APs as autophagy induction in AD brain remains high^29^. However, as accumulating AVs deplete sources of available membrane, APP-rich ER and Golgi membranes join endosomes as major sources of APP-βCTF/Aβ generation. Therefore, ER and Golgi are likely key contributors to the expansion of the amyloid fibril network, supporting AP/AL formation by contributing both membrane and β-amyloid precursor.

**Fig. 7:**
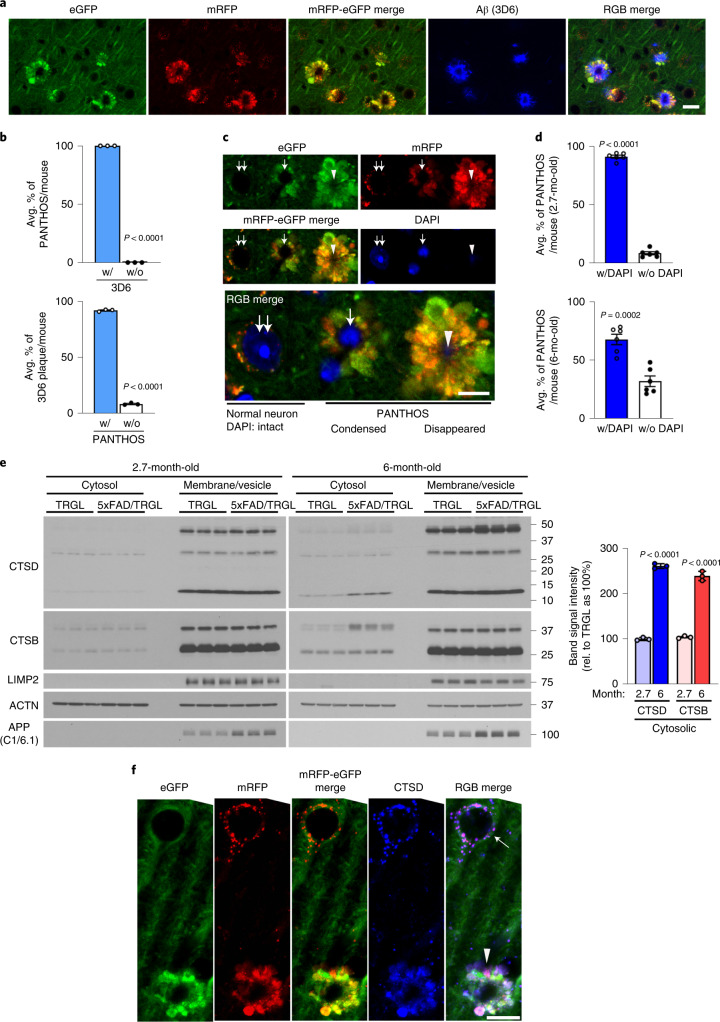
PANTHOS neurodegeneration coincides with β-amyloid plaque formation and subsequent lysosomal neuronal cell death. **a**, Aβ antibody 3D6 detecting the appearance of amyloid plaques in 5xFAD mice (2.7-month-old male) demonstrates co-incidence with the presence of a PANTHOS neuron. Scale bar, 20 μm. **b**, Quantitative percentage of PANTHOS neurons that are 3D6-positive (top) and percentage of PANTHOS among 3D6-positive plaques that are associated with PANTHOS (bottom)—with PANTHOS (91.7 ± 0.5%), without PANTHOS (8.3 ± 0.5%), with 3D6 (100 ± 0%), without 3D6 (0 ± 0%). *n* = 3 mice. **c**, DAPI staining depicting various stages of PANTHOS development and ultimate disappearance of detectable DAPI (although not necessarily nuclear marker IR; see Extended Data Fig. 5e). Normal DAPI-labeled nucleus (double arrow), condensed DAPI signal (single arrow) and non-detectable DAPI in very advanced PANTHOS neuron (arrowhead). Scale bar, 10 μm. **d**, Percentage of PANTHOS neurons with detectable DAPI label in 2.7-month-old or 6-month-old 5xFAD/TRGL mouse brain. 2.7 months: with DAPI (91.4 ± 1.3%) and without DAPI (8.6 ± 1.3%); 6 months: with DAPI (67.8 ± 4.5%) and without DAPI (32.2 ± 4.5%). *n* = 6 (two sections per mouse, three mice; 94 neurons in cortex area were counted). **e**, Lysosomal enzyme distribution in cytosol and membrane/vesicle fraction in 2.7-month-old and 6-month-old 5xFAD and WT male mouse cortex. Cytosolic CTSD: 2.7 months (99.8 ± 1.9%) and 6 months (260.4 ± 3.1%); cytosolic CTSB: 2.7 months (103.8 ± 1.6%) and 6 months (238.5 ± 5.9%). *n* = 3 mice per each genotype. **f**, Immunofluorescence labeling of 2.7-month-old 5xFAD/TRGL mouse brain neurons with a CTSD antibody. Arrow indicates normal CTSD-positive puncta in a healthy neuron. The experiment was repeated three times independently with similar results. The arrowhead indicates diffuse CTSD signal in a PANTHOS neuron. Scale bar, 20 μm. Quantitative data are presented as means ± s.e.m., unpaired *t*-test, two-tailed *P* value as indicated. mo, month; rel., relative. Source data

Consistent with PANTHOS being the principal source of amyloid plaques, immunolabeling of β-amyloid with 3D6 in 5xFAD/TRGL mice revealed an exclusive co-incidence and a one-to-one quantitative relationship between individual PANTHOS neurons and individual amyloid plaques (Fig. 7a). All PANTHOS neurons were 3D6-positive, and 91.7 ± 0.01% of the total 3D6 signal in brain was detectable in PANTHOS lesions (*n* = 3 mice, 105 neurons and 94 lesions counted) (Fig. 7b). Moreover, a DAPI-positive nuclear signal, including condensed or fragmented/diffuse signals in the perikaryal center (Fig. 7c), was detectable in 91.4 ± 1.29% (*n* = 6, two sections per mouse) of PANTHOS lesions in cortex from 2.7-month-old 5xFAD/TRGL mice (Fig. 7d, top graph). In older mice (6 months), 67.8% of the PANTHOS neurons still displayed DAPI nuclear signal (Fig. 7d, bottom graph) despite glial invasion and advanced neurodegeneration. This percentage is likely an underestimate because immunoEM analyses with the nuclear marker KDM1/LSD1 revealed nuclear remnants even after loss of nuclear integrity (Extended Data Fig. 5e). The temporal and 1:1 spatial relationship among PANTHOS, intracellular perinuclear Aβ accretion and amyloid plaque formation, therefore, indicates that the vast majority of amyloid plaques originate from a corresponding individual PANTHOS neuron. The transition from intact nucleated PANTHOS neurons to the more advanced stage of DAPI disappearance with glial invasion of the cell likely represents the loss of cellular integrity and conversion to an extracellular plaque.

### Lysosomal permeabilization promotes neuronal cell death

**Extended Data Fig. 7:**
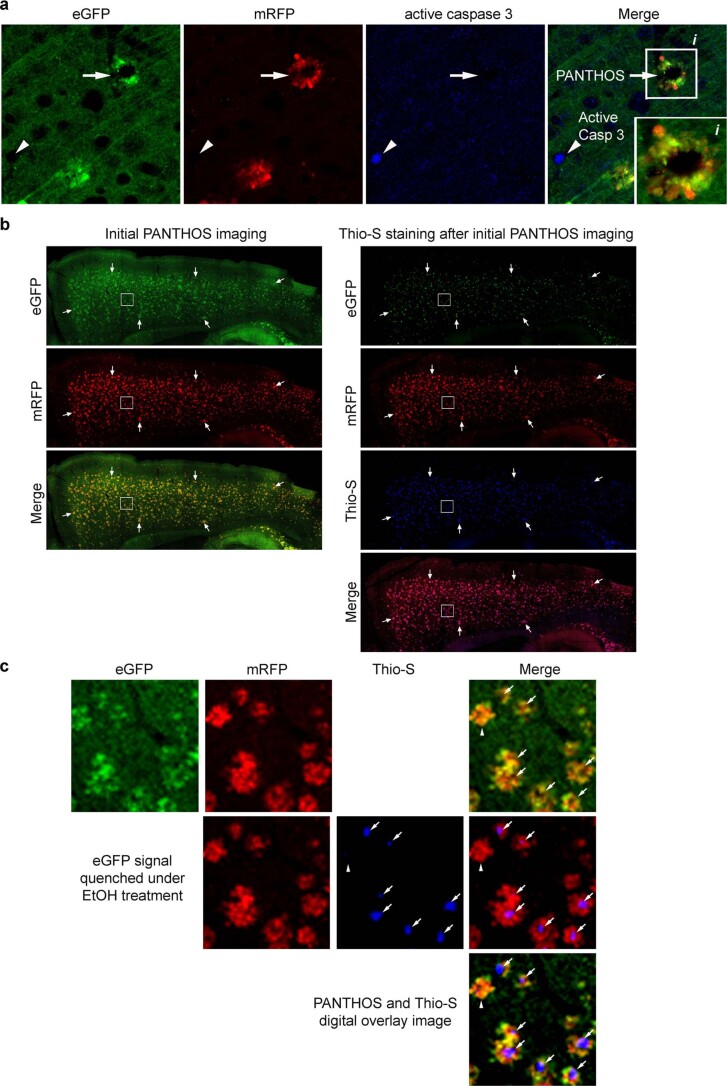
PANTHOS neurons evolve into Thio-S positive dense-cored senile plaques in the 5xFAD/TRGL AD mouse model. **a**. PANTHOS neurons are not positive for the anti-active caspase-3 antibody in 2.7-month-old, male 5xFAD/TRGL mouse brain. Although active caspase-3 positive cells were extremely rare and did not overlap with PANTHOS, the arrowhead identifies a rare non-neuronal caspase-3-positive cell as a positive control. **b**. Representative image of PANTHOS with GFP/RFP filter set (left) and image of the additional Thio-S staining with GFP/RFP/DAPI filter in 6-month-old, male 5xFAD/TRGL mouse brain. eGFP signal of the PANTHOS was diminished, whereas mRFP signals were preserved in Thio-S-stained tissues (right) compared to unstained tissue (left). Arrow used as tissue orientation. **c**. Digital overlay of the ROI (Fig. b, box) highlights that PANTHOS profiles are only detectable using mRFP signal since fixation for Thio-S quenches GFP. A small percentage of PANTHOS were Thio-S negative (arrowhead) whereas the majority are Thio-S positive (arrow) in the cortex of 6-month-old, male 5xFAD/TRGL mouse brain. **a-c**: Experiment was repeated 3 times independently with similar results.

Lysosomal alkalinization is reported to promote lysosomal membrane permeabilization and cathepsin release into cytosol^30^. Cytosolic and membrane/vesicle fractionation analyses markedly increased levels of lysosomal enzymes in the cytosol of brain from 6-month-old 5xFAD mice compared to brains from WT littermates (Fig. 7e). Lysosomal enzyme leakage was detectable at 6-month, but not young (2.7-month), brains, when many fewer neurons are affected. We further examined the association of PANTHOS with lysosomal membrane permeabilization using CTSD IHF. Compared to an adjusted normal neuron (Fig. 7f, arrow), a PANTHOS neuron (Fig. 7f, arrowhead) displayed diffuse CTSD immunoreactivity in a 5xFAD/TRGL mouse brain co-labeled with CTSD. We ruled out the involvement of a caspase-3-mediated apoptotic cell death, because PANTHOS neurons were caspase-3-negative (Extended Data Fig. 7a).

### PANTHOS neurons evolve into senile plaques in AD models

**Fig. 8:**
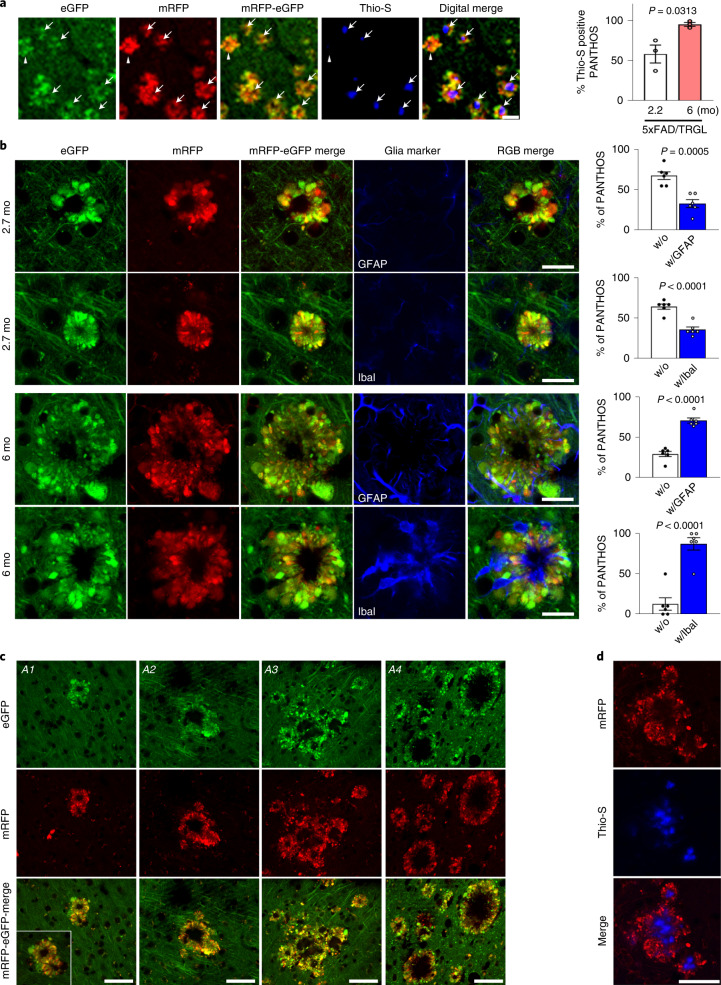
PANTHOS neurons evolve into classical dense-cored senile plaques in AD models. **a**, Dense-cored senile plaque labeling using Thio-S in 2.2-month-old or 6-month-old 5xFAD/TRGL mice. Quantified presence of Thio-S within the confines of a PANTHOS neuron (*n* = 3 mice). 2.7 months (58.1 ± 11.2%) and 6 months (95.2 ± 2.4%). Scale bar, 50 μm. See also Extended Data Fig. 7. **b**, IHF labeling using markers of astrocytes (GFAP) or microglia (Iba I) in 2.7-month-old or 6-month-old 5xFAD/TRGL mice. Quantified presence of microglia or astrocytes within the confines of a PANTHOS neuron. 2.7 months: without GFAP (67.2 ± 4.8%), with GFAP (32.8 ± 0.8%), without IbaI (64.2 ± 3.1 %), with IbaI (35.8 ± 3.1%); 6 months: without GFAP (29.3 ± 3.2%), with GFAP (70.7 ± 3.2%), without IbaI (12.6 ± 7.7%), with IbaI (87.4 ± 7.7%). *n* = 6 (two sections per mouse, three mice). Scale bar, 20 μm. **c**, Growth of a senile plaque commonly occurs by coalescence of one or multiple adjacent PANTHOS neurons and the progressive clearance of cellular debris after centrally located cells have degenerated, leaving behind the poorly degradable amyloid originating from these neurons. A1–A3: 12-month-old Tg2576/TRGL; A4: 25.5-month-old APP51/TRGL mouse brain. Scale bar, 50 μm. See also Extended Data Fig. 8. **d**, Growth of a Thio-S-positive dense-cored senile plaque commonly occurs by coalescence of one or multiple adjacent PANTHOS neurons. Scale bar, 50 μm. **c**, **d**, The experiment was repeated three times independently with similar results. Quantitative data are presented as means ± s.e.m., unpaired *t*-test, two-tailed *P* value as indicated. mo, month. Source data

To characterize the evolution of PANTHOS neuron lesions into mature plaques, we immunolabeled PANTHOS with Thioflavin S (Thio-S) for the detection of dense-cored senile plaques (Fig. 8a and Extended Data Fig. 7b,c). In quantitative analyses of 5xFAD/TRGL at 2.2 months of age, half of the PANTHOS profiles were Thio-S-positive, whereas, in 6-month-old 5xFAD/TRGL mice, more than 95% were Thio-S-positive (Fig. 8a, graph). To further characterize the evolution of PANTHOS neuron lesions into mature plaques, we immunolabeled reactive astrocytes and microglia. Neither glial cell type was frequently associated initially with PANTHOS neurons, and, therefore, these cells were unlikely to be a major triggering factor in PANTHOS development. In quantitative analyses of 5xFAD/TRGL at 2.7 months of age, most PANTHOS neurons were unengaged by microglia or astrocytes (Fig. 8b). In older 5xFAD/TRGL mice (6 months), when greater numbers of PANTHOS neurons exhibited advanced loss of structural integrity, relatively few affected neurons were unengaged by microglia and astrocytes (Fig. 8b).

**Extended Data Fig. 8:**
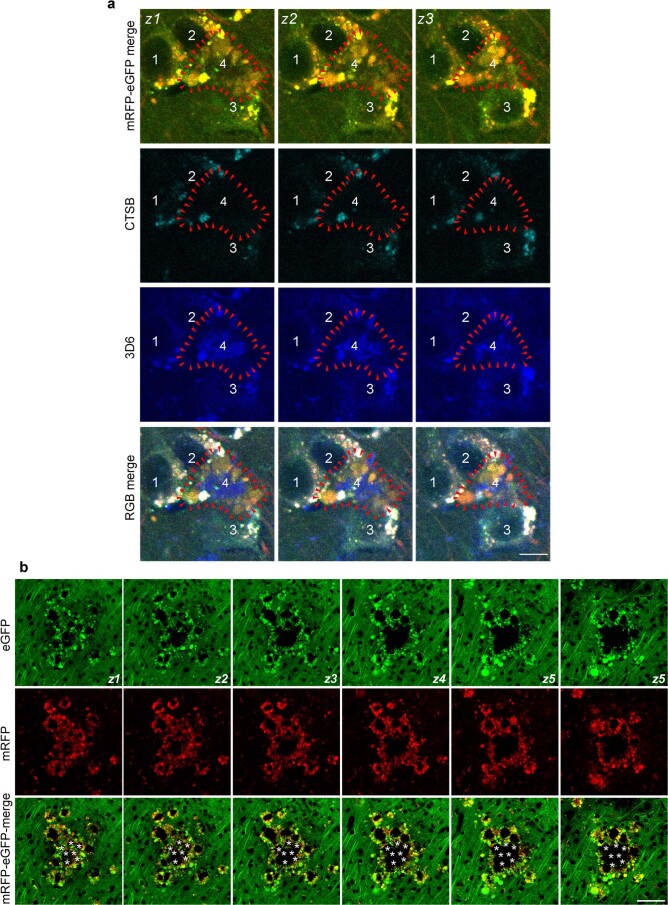
Recruitment of degenerating cells and individual PANTHOS coalescence in old APP51 mice. **a**. IHF co-labeling of 28-month-old APP51/TRGL mouse brain layer V cortical neurons with LY marker (CTSB) and 3D6 monoclonal antibody against APP-βCTF/Aβ. A Z-stack series (1 μm-thick, *z*1-*z*3) shows recruitment of various cells (1-3) with different degrees of perikaryal pa-AL and 3D6 accumulation around an amyloid-invaded PANTHOS neuron (4, arrowheads). Scale bar 10 μm. **b**. Serial z-stacked image (1 μm thick, number *z1- z5*) showing multiple single PANTHOS become united into one large structure. * Denotes trace of the individual PANTHOS. Scale bar 50 μm. **a-b**: Experiment was repeated 3 times independently with similar results.

In older 5xFAD mice, PANTHOS lesions frequently expanded into larger senile plaques when adjacent PANTHOS neurons merged into a single larger structure (Fig. 8c, A1 and A2, respectively) that comprised multiple Thio-S-positive dense cores (Fig. 8d). Within these growing lesions, newly recruited PANTHOS neurons could still be recognized (Extended Data Fig. 8a, arrowheads), but loss of integrity of the original PANTHOS neurons and its adjacent neighbors created an expanding central core of persisting β-amyloid as other cellular debris is cleared, yielding, finally, an enlarged extracellular dense-cored senile plaque (Fig. 8b, A3 and A4, respectively), as confirmed by *z*-stack confocal imaging (Extended Data Fig. 8b).

## Discussion

**Extended Data Fig. 9:**
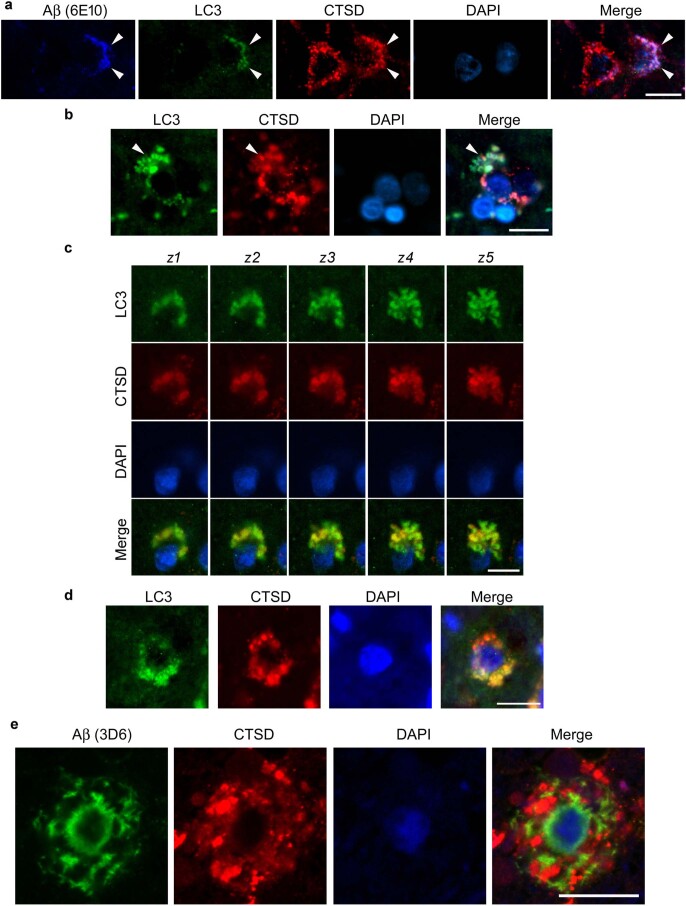
Autophagy-Lysosomal Pathway (ALP) abnormality in Braak II stage) human AD brain degenerating neuron. **a**. Representative fluorescence images of intraneuronal Aβ in autolysosomes (arrowhead, autophagy (LC3)/lysosomal (CTSD)) together with DAPI for nucleus. Scale bar 20 μm. **b**. Representative LC3/CTSD fluorescence images depicting a neuron with focal plasma membrane blebbing as pa-AL enlarge and proliferate (arrowhead). **c**. Z-stacked image series (1 μm thick, number *z1*–*z5*) showing LC3 and CTSD positive blebs emanating from perikaryon marked by DAPI staining. Scale bar 10 μm. **d**. Patterns of AV-related pathology showing a neuronal perikaryon with an intact nucleus. Enlarged LC3- and CTSD-positive vesicles (AL) are contained within numerous perikaryal membrane blebs Scale bar 10 μm. **e**. IHF labeling of Aβ (4G8) and DAPI stain. Perinuclear intraneuronal Aβ accumulation surrounding visible DAPI-positive nucleus within a PANTHOS like neuron. Scale bar 10 μm. **a-d**: Experiment was repeated 3 AD human brain independently with similar results.

**Extended Data Fig. 10:**
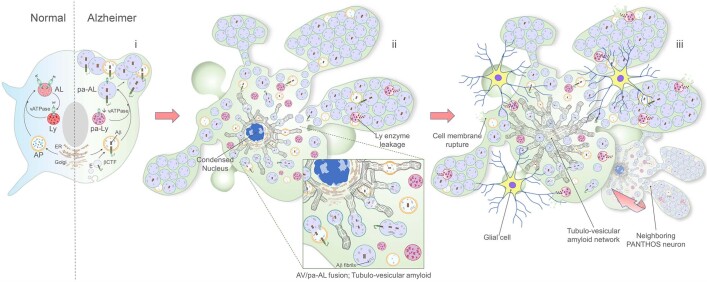
Diagram summarizing the stages of autophagy-lysosomal pathway-mediated PANTHOS (“poisonous flower”) neurodegeneration in AD mice. Normal autophagic clearance involves substrate sequestration into a double-membrane autophagosome (AP) followed by fusion with lysosomes (LY), yielding a single-membrane autolysosome (AL). The proton pump vATPase maintains an acidic pH (4.5-5) optimal for lysosomal enzymatic activity and degradation of substrates within AL, which then convert to lysosomes to restore normal levels of free LY. In Alzheimer’s disease, three main stages of neuronal compromise and degeneration resulting from autophagy-lysosomal pathway dysfunction can be identified: **i) The “budding” stage of PANTHOS: AL acidification deficiency and poorly acidified-AL build-up**. AD-gene driven deficits of Ly vATPase activity underlie impaired clearance of autophagic substrates, including APP-βCTF/Aβ (mainly derived from the endolysosomal pathway). The result is an accumulation of enlarged poorly acidified AL (pa-AL) within the neuronal perikaryon well before the appearance of any other overt AD-related pathology. Buildup of pa-AL containing APP-βCTF/Aβ is accompanied by their progressive peripheralization resulting in plasma membrane distortion and bulging/budding (*see* Fig. 3a, f; Fig. 4a and Fig. 6a). **ii) The “flowering” stage of PANTHOS: formation of perinuclear membrane-bound amyloid fibers**. Massive buildup of APP-βCTF/Aβ-containing pa-AL induces a unique pattern of perikaryal membrane blebbing. The blebs, corresponding to the “petals” of the PANTHOS neuron, have tapered necks extending toward the plasma membrane-surface of the PANTHOS neuron containing a degenerating condensed nucleus (*see* Fig. 4a-e; Fig. 5a and Fig. 6b-c). β-amyloid (Aβ) fiber bundles within a branching membrane tubular network accumulate around a deteriorating nucleus and reflect the fusion of AVs with APP-rich endoplasmic reticulum (ER). The enlarged inset shows AVs at different stages of fusion (*see* Fig. 5c,d and Fig. 6d,e). Accrual of Aβ and other oxidized substrates initiates Lysosomal Membrane Permeabilization (LMP) and LY enzyme leakage. **iii) The “overblown” stage of PANTHOS: amyloid plaque expansion via glial invasion and recruitment of neighboring PANTHOS neurons**. As nuclear membrane is disrupted and the nucleus degenerates, amyloid fiber growth within the expanding perinuclear membrane-tubular network, completely invades the center of the PANTHOS neuron incorporating additional AVs (*see* Fig. 5c; Extended Data Fig. 5e and Fig. 6e). LY enzyme leakage, along with focal rupture of perikaryal and bleb plasma membrane, trigger an inflammatory response and signals that recruit phagocytic glial cells and promotes the coalescence of individual PANTHOS neurons, which expand the plaque lesion and central protease-resistant β-amyloid core (*see* Fig. 7e,f and Fig. 8a,b,c), transforming degenerating PANTHOS perikarya into an extracellular senile plaque.

Our transgenic dual-fluorescence probe, identifying autophagic compartments and associated changes in their pH in vivo, established that autophagy failure in five different APP-AD mouse models originates from an early decline of AL/LY acidification. Furthermore, we uncovered a previously undescribed pattern of extreme autophagic stress, termed PANTHOS, in individual neuronal perikarya, which is characterized by massive perikaryal accumulations of poorly acidified AVs containing APP-βCTF/Aβ. The advance of PANTHOS generates an intraneuronal perinuclear ‘core’ of β-amyloid within membrane tubules. Preliminary analyses of human AD brain (Extended Data Fig. 9) revealed a similar PANTHOS in selected neocortical neurons, which is most easily appreciated immunocytochemically at the Braak II pathologic stage as the first β-amyloid plaques are forming. Progressive failure of an initially neuroprotective autophagy response in neurons^29^ is accompanied by an evolution of PANTHOS toward neuronal cell death involving lysosomal membrane permeabilization, cathepsin release and, ultimately, glial invasion and extracellular plaque formation and expansion (Extended Data Fig. 10, diagram).

AL acidification deficits in Tg2576 mice were detectable by 5 months of age—more than 4 months before β-amyloid deposited extracellularly. The emergence of pH deficits in AL coincided with lowered vATPase activity in brain LYs—the likely molecular basis for the acidification deficit. Declining AL acidification was accompanied by selective build-up of APP-βCTF and Aβ within enlarged pa-ALs. These APP metabolites are known to be both generated and degraded in ALs^12,31^ and amphisomes^32^. We also cannot exclude additional delivery of APP-βCTF and Aβ to AL/LY through microautophagy and chaperone-mediated autophagy^33^.

The exceptional resolution of our tfLC3 autophagy probe, combined with advanced ultrastructural and multiplex confocal imaging methods, enabled the recognition of a unique pathobiologic process (‘PANTHOS’) in intact neurons within vulnerable cell populations. This morphologic pattern, not previously reported, to our knowledge, includes AV accumulation (‘autophagic stress’) so extreme that it induced huge AV-filled plasma membrane blebs and accelerated perinuclear accretion of Aβ and β-amyloid fibrils within tubulo-vesicular structures created, in part, through AV fusion. Large AV-filled blebs were shown by CLEM and 3D serial ultrastructural analyses to be formed by plasma membrane evagination and to originate from the perikaryal cytoplasm. Their asymmetric morphology, high hydrolase content and extensive distribution encircling an affected perikaryon far outnumbered DNs. Unlike perikaryal blebs, DNs were commonly enriched with neurofilaments, only weakly cathepsin-immunoreactive and LAMP2-immunoreactive and frequently myelinated.

Autophagic stress develops in many congenital lysosomal storage disorders (LSDs). In several of these disorders, Niemann–Pick type C (NPC1)^34^ and mucopolysaccharidosis type III (MPS-III), pH has been shown to be elevated^35^. Among LSDs, NPC1 has considerable phenotypic overlap with AD (for example, paired-helical filaments, cholinergic neurodegeneration, endosome anomalies, disease acceleration by ApoE4, intracellular Aβ/βCTF elevation and modest amyloid deposition^36–38^); tauopathy has been reported in mouse MPS-III models and intracellular synuclein, and Aβ accumulations are detected in MPS-III brain^39^. That individuals with these disorders usually do not survive to mid-adult ages may partly explain the infrequency of amyloid plaques. Even individuals with AD due to *PSEN1* mutations generally do not develop amyloid plaque pathology until the fourth decade of life, when aging factors may contribute^10,40^. Also, neuronal ALP induction continues to increase in AD brain even as substrate clearance declines^29,41^, compounding autophagic stress and likely increasing βCTF/Aβ generation^12^.

PANTHOS neurons account quantitatively for the overwhelming majority of plaques that developed in five different AD models at the ages studied. In young 5xFAD mice, β-amyloid cored plaques, detected with β-amyloid antibodies, showed nearly 1:1 coincidence with a single PANTHOS neuron exhibiting a central nucleus. Even at a relatively late stage of compromise, intracellular Aβ-immunoreactive fibrils forming a perinuclear core are mainly contained within membrane-bound tubular structures derived from fusion of Aβ-positive ALs. This stage can be reached without appreciable microglial or astrocytic invasion that would reflect ‘eat me’ signaling from dying neurons^42,43^, implying, therefore, that neuronal structural integrity is prolonged even as PANTHOS is quite advanced. Subsequent microglial and astrocytic invasion of the PANTHOS neuron heralds the eventual cell death that converts this amyloid lesion within an intact neuron into an extracellular amyloid plaque.

β-amyloid plaque formation in AD has commonly been considered to originate from extracellular deposition of β-amyloid derived from secreted Aβ, which then triggers secondary neuritic dystrophy and neuronal cell death. By contrast, our evidence in diverse AD models supports the opposite sequence—namely, extracellular plaques mainly evolve from intraneuronal build-up of β-amyloid within membrane tubules, forming a centralized amyloid ‘core’ within single intact PANTHOS neurons that subsequently degenerate to give rise to the classical senile plaque. This ‘inside-out’ process accords with and substantiates hypotheses from many investigators^44,45^. In versions of this hypothesis, Aβ and its oligomeric species generated intracellularly within ALP compartments can gain access to the extracellular space by neurodegeneration, local membrane damage or unconventional secretion (exocytosis). Importantly, a few investigators have described intracellular membrane-enclosed amyloid fibrils in AD mouse models^46^ and, in AD brain, the frequent presence of amyloid surrounding DAPI-positive nuclei^47,48^ and neuronal lysosomal hydrolase abundance within extracellular β-amyloid^49^.

Our findings add to mounting evidence that lysosomal acidification and the dysregulation of the vATPase complex are common targets of genetic and metabolic disruptions associated with neurodegenerative disease^50^. Coupled with previous evidence^10,20^, our findings strongly support a pathogenic link between APP metabolites and LY dysfunction in AD. Notably, remediating *PSEN1*-related lysosomal pH deficits by various means ameliorates autophagy failure and other AD-related pathology in AD models^7,24^. Additional supporting evidence from our group shows that the PANTHOS cascade in APP-based AD models described in this report can be significantly alleviated by pharmacologically targeting the lysosomal pH deficit. Beyond the significance of findings revealed here, we anticipate broad potential of our transgenic dual-fluorescence tfLC3 autophagy probe to characterize ALP changes sensitively over time in other neurodegenerative disease models and to facilitate assessment of autophagy/lysosome modulators as therapeutic agents.

## Methods

### Cell lines and reagents

WT and APPswe stably expressed murine neuroblastoma (N2a) cells were maintained in DMEM with penicillin–streptomycin and 10% FBS at 37 °C and 5% CO_2_ (ref. ^51^).

### Mouse lines and animal care

We used the Tg2576 mouse line (B6;SJL-Tg(APPSWE)2576Kha), which expresses mutant human APP (Swedish K670N/M671L) and is maintained on a B6;Dba/2F1;SW background. For TRGL (**T**hy-1 m**R**FP-e**G**FP- **L**C3) mouse generation, targeting vector for tfLC3 was constructed by insertion of tfLC3 into Thy1.1 expression cassette^13,52^. Tg2576/TRGL mice were studied at 1.6, 5, 10 and 12 months together with TRGL littermates as a control. The tfLC3 was crossed with 5xFAD (Tg6799, C57BL/6NTAC), which expresses mutant human *APP* and *PSEN1* (APP KM670/671NL: Swedish, I716V: Florida, V717I: London, PSEN1 M146L, L286V)^53^, and then tfLC3/5xFAD mice were studied at 1.6, 2.7, 4 and 6 months together with age-matched controls. TgCRND8 mice, which express mutant human APP (Swedish K670N/M671L and Indiana V717F)^54^, were crossed with TRGL, and 1.9-month-old males were used. PS/APP mice^55^, which express mutant human APP (Swedish K670N/M671L) and mutant PS1 (PS1M146L), were crossed with TRGL, and 3.1-month-old males were used. APP51 mice^23^, which express WT human APP751, were crossed with TRGL, and females were used. Detailed mouse age and sex information are in the figure legends. The mice were maintained in the Nathan Kline Institute (NKI) animal facility and housed at ~22.8 °C room temperature with a humidity level of ~55% and on a 12-hour light/dark cycle. All animal experiments were performed according to the ‘Principles of Animal Care’^56^ and approved by the Institutional Animal Care and Use Committee at the NKI.

### Human brain

Paraformaldehyde (PFA)-fixed tissue blocks obtained from prefrontal cortex (Brodmann area 9/10) were kindly provided from Emory Alzheimer’s Disease, from Marla Gearing (Alzheimerʼs Disease Research Centers/Center for Neurodegenerative Disease), with demographic information outlined.

We used Braak stage II brains (E05-57: 86 years old, black female with postmortem interval (PMI) of 6 hours; E05-54: 85 years old, white female with PMI of 7 hours; OS96-08: 65 years old, white male with PMI of 4 hours).

### Antibodies and reagents

Anti-PS1 loop mouse monoclonal antibody (MAB5232:clone PS1-loop, 1:1,000) and anti-nicastrin mouse monoclonal antibody (MAB5556: clone 9C3, 1:1,000) were purchased from Chemicon. Rabbit anti-CTSD (Rudy4, 1:2,000) antibody and NFL (21.4, 1/250) were produced in-house^18^. CTSB was from Neuromics (GT15047, 1:250). LAMP2 was from the Developmental Studies Hybridoma Bank (ABL-93, 1:200). LIMP2 was from Novus (NB400-129, 1:200). Antibodies directed against APP, Aβ and/or other APP proteolytic species included APPc (Sigma-Aldrich, A8717, 1:250); 4G8 (BioLegend: clone 4G8, 800701, 1:250); and C1/6.1 monoclonal antibody against the C-terminal 20 residues of APP (made in-house, 1:400, NKI). Additional mouse monoclonal antibodies were generous gifts from Marc Mercken (Janssen Pharmaceuticals/Johnson & Johnson): JRF/AβN/25 (specific to Aβ1-7, 1:200); 3D6 (specific to Aβ1-5, 1:250); JRF/cAb42/26 (specific to Aβ42, 1:200)^57^; MAP2 (Sigma-Aldrich, M9942: clone HM-2, 1:250); NSE (Dako, M0873: clone BBS/NC/VI-H14, 1:250); and histone H3 (4499, 1:200). Lamin A/C (4777: clone 4C11, 1:200) and Tom20 (42406, 1:2,000) were from Cell Signaling Technology. KDM1/LSD1 (Abcam, ab129195: clone EPR6825), GFAP (Sigma-Aldrich, AB5804, 1:250), IbaI (Wako, 019-19741, 1:250), ATP6 V1A (GeneTex, GTX110815, 1:1,000), ATP6 V0a1 (Abcam, ab176858, 1:2,000) and Rab5 (Abcam, ab218624: clone EPR21801, 1:1,000). Rab7 (Cell Signaling Technology, 9367: clone D95F2, 1:1,000), PDI (BD Biosciences, 610946: clone 34, 1:1,000), STX6 (Cell Signaling Technology, 2869: clone C34B2, 1:2,000), Tubulin (Sigma-Aldrich, T8535:clone JDR.3B8, 1:5,000), Actin (Sigma-Aldrich, A1978: clone AC-15, 1:5,000) and anti-p62 (ProGen Biotech, GP62-C, 1:500). Anti-SEC61B rabbit pAb (15087-1-AP, 1:1,000) was from Proteintech. HRP-linked rabbit IgG (711-035-152, 1:5,000), mouse IgG (711-035-150, 1:5,000), rat IgG (712-035-150) and goat IgG (705-035-003) secondary antibodies were purchased from Jackson ImmunoResearch. Prolong Diamond Antifade Mount (P36961), goat anti-mouse Alexa Fluor 647 (A21235), goat anti-rat Alexa Fluor 647 (A21247), goat anti-rabbit Alexa Fluor 647 (A21245) and donkey anti-rabbit Alexa Fluor 405 (A48254) secondary antibodies were from Thermo Fisher Scientific. Mouse on Mouse (M.O.M) detection kit (BMK-2201), normal-donkey (S-2000-20) and normal-goat (S-100) serum blocking solution were from Vector Laboratories. Thio-S (T1892) was from Sigma-Aldrich.

### Ratiometric analysis of AL and AP acidity

Procedures were performed as previously described^13^. Confocal images were analyzed with the Zen Blue Image Analysis Module from Carl Zeiss Microscopy. The R, G and B intensity values of each vesicle were calculated using the profile function of Zen. The RGB ratio of each vesicle was converted into a hue angle and saturation range by entering the values of R, G and B for a given puncta into the formula as follows: Hue° = IF(180/PI()×ATAN2(2×R-G-B,SQRT(3)×(G-B)) < 0,180/PI()×ATAN2(2×R-G-B,SQRT(3)×(G-B)) + 360,180/PI()×ATAN2 (2×R-G-B,SQRT (3) × (G-B))). Saturation percent of the hue angle was calculated by entering the values of R, G and B for a given puncta into the following formula = (MAX(RGB) − MIN(RGB)) / SUM(MAX(RGB) + MIN(RGB))×100, provided lightness is less than 1, which is the usual case for our data. Hue angle was converted to color using the hue color wheel.

### Subcellular fractionation, gel electrophoresis and western blotting

AV prep: Procedures were performed as previously described^58^. For each mouse genotype, cerebral cortices from five or more brains were pooled. The samples were homogenized and subjected to differential centrifugation to separate a fraction enriched in AVs, LYs and mitochondria as previously described. The different organelles in this fraction were isolated by floatation in a discontinuous gradient of metrizamide (50%, 26%, 24%, 20% and 10%), and the LY-enriched fraction was recovered in the 24–16% interface. A fraction enriched in ER resealed vesicles (microsomes), and the cytosolic fraction was obtained in the pellet and supernatant, respectively, after centrifugation of the supernatant at 100,000*g* for 1 hour. Cytosol and membrane/vesicle prep: Cerebral cortices from male 5xFAD and WT mouse brain were homogenized with buffer (20 mM Tris-Cl, pH 7.4 with 250 mM sucrose, 1 mM EGTA, 1 mM EDTA, 1 mM MgCl_2_ and protease and phosphatase inhibitor (Roche)). The post-nuclear homogenates obtained by centrifugation (1,000*g*, 10 minutes) were further fractionated into cytosolic and membrane/vesicle fractions by high-speed centrifugation (150,000*g*, 50 minutes), and equal proteins were loaded on a gel. Samples were mixed with 2× SDS sample buffer and incubated for 5 minutes at 100 °C. After electrophoresis on a 4–20% Tris-glycine gradient gel (Invitrogen), proteins were transferred onto 0.45-µm PVDF membranes (Millipore) for detection of all other proteins and then incubated overnight in primary antibody. HRP-conjugated secondary antibody was added the next morning and incubated for 1 hour at room temperature. The blot was developed using an Invitrogen ECL kit.

### vATPase activity assay

AD transgenic mice were studied at the indicated age point together with WT or TRGL littermates as a control. Mouse hemi-brain was homogenized in 10× volume of homogenization buffer by 40 strokes in a Teflon-coated pestle. Lysates were centrifuged at 1,000*g* for 20 minutes to generate the post-nuclear supernatant (PNS). The PNS was then adjusted to 25% OptiPrep (Sigma-Aldrich, D1556) with 50% OptiPrep in HB. The resulting mixture, 2 ml in 25% OptiPrep, was placed at the bottom of a clear ultracentrifuge tube (14 × 95 mm, Beckman Coulter) and was overlaid successively with 1.5 ml each of 20%, 15%, 14%, 12.5%, 10% and 5% OptiPrep in cold HB. The gradients were centrifuged for 18 hours at 100,000*g* at 4 °C in an SW 40 rotor (Beckman Coulter). Next, 500-μl fractions were collected from the top of the ultracentrifuge tubes and analyzed by WB analysis. LY-enriched fractions (mixture of 20 μl of each OptiPrep fraction from 15 to 18) were mixed with 0.052% NaN_3_ for blocking the mitochondrial ATPase activity. The vATPase activity was measured using the ATPase Assay Kit (Innova Biosciences, 601-0120) according to the manufacturer’s protocol. Control samples were measured in the presence of the vATPase inhibitor concanamycin A (1 μM) (Sigma-Aldrich, C9705), and the experimental values were subtracted accordingly. Absorbance was measured at 650 nm, and solutions of P_i_ were used to generate a standard curve.

### Ultrastructural EM analyses

Mice were perfused with 2.5% glutaraldehyde and 2% PFA in 0.1 M sodium cacodylate buffer, pH 7.4 (Electron Microscopy Sciences). Brains were removed and sectioned using a vibratome into 50-µm or 100-µm sections and placed in fixative solution and stored at 4 °C. Samples were then treated with 1% osmium tetroxide in 100 mM sodium cacodylate buffer pH 7.4 for 30 minutes, washed in distilled water four times (10 minutes per wash) and then treated with 2% aqueous uranyl acetate overnight at 4 °C in the dark. Samples were then washed and sequentially dehydrated with increasing concentrations of ethanol (20%, 30%, 50%, 70%, 90% and 100%) for 30 minutes each, followed by three additional treatments with 100% ethanol for 20 minutes each. Samples were then infiltrated with increasing concentrations of Spurr’s resin (25% for 1 hour, 50% for 1 hour, 75% for 1 hour, 100% for 1 hour and 100% overnight at room temperature) and then incubated overnight at 70 °C in a resin mold. For transmission electron microscopy ultrastructural analysis, 70-nm sections were cut using a Leica Reichert Ultracut S ultramicrotome and a Diatome diamond knife, placed onto grids and then post-stained with 2% uranyl acetate and lead citrate. Images were taken using a Ceta camera on a Thermo Fisher Scientific Talos L120C transmission electron microscope operating at 120 kV.

For the 100-µm-thick embedded samples for serial block-face scanning electron microscopy (SEM), a diamond wire saw was used to remove excess resin around the embedded tissue. The trimmed block was then glued to a VolumeScope specific SEM stub (Agar Scientific, AGG1092450) using a two-part silver conductive epoxy (Ted Pella, H20E EPO-TEK). The sample was further trimmed down to a block face of 1,000 µm × 900 µm and 400 µm deep using an ultramicrotome. Only the slides of the block were sputter coated with a 30-nm-thick layer of gold, as the bottom of the block was already mounted to the stub with the silver conductive epoxy before gold coating, and the top of the block was covered during the coating process.

The final prepared sample was imaged using an Apreo scanning electron microscope (Thermo Fisher Scientific) equipped with a VolumeScope module for serial block-face imaging operating in low vacuum mode at 50 Pa using a pole piece mounted backscatter detector, VS-DBS. The brain tissue (M-A) dataset was acquired using an accelerating voltage of 2 kV and a beam current of 100 pA. A total of 509 images were collected with a slice cutting thickness of 100 nm. The final image dimension was 8,855 × 9,500 with a pixel resolution of 15 nm in *x* and *y* and a dwell time of 5 µs.

### ImmunoEM and acid phosphatase histochemistry

Tissue was processed as described above. Sections of 70 nm were cut on a Leica ultramicrotome with a diamond knife. The sections were placed onto carbon formvar 75 mesh nickel grids and etched using 4% sodium metaperidotate for 10 minutes before being washed twice in distilled water and then blocked for 1 hour. Grids were incubated with 3D6, KDM1/LSD1, LC3 or CTSD antibodies (1:2 dilution) at 4 °C overnight. The next day, grids underwent seven washes in 1× PBS and were then incubated in anti-mouse or anti rabbit 10-nm gold secondary (1:50 dilution) for 1 hour. After this, the grid was washed seven times in 1× PBS and twice in distilled water. Grids were then silver enhanced for 5 minutes (Nanoprobes). Grids were finally post-stained with 1% uranyl acetate for 5 minutes, followed by two washes in water and then stained with lead citrate for 5 minutes, followed by a final two washes in distilled water. Samples were then imaged on a Thermo Fisher Scientific Talos L120C operating at 120 kV. Acid phosphatase histochemistry: PS/APP mouse brains were transcardially perfused with fixative (4% PFA, 1% glutaraldehyde in 0.1 M sodium cacodylate buffer, pH 7.4, containing 0.025% calcium chloride, 5% sucrose and 0.075% cytidine 5′-monophosphate (CMP)). The brains were removed and further immersion-fixed in 4% PFA for 4 hours at 4 °C. Vibratome sections (50 µm) were cut, rinsed in 0.1 M sodium cacodylate buffer containing 5% sucrose and then in 0.05 M Tris-maleate buffer containing 5% sucrose, followed by incubation in the reaction medium (25 mg of CMP, 7 ml of distilled water, 10 ml of 0.05 M Tris-maleate buffer with 5% sucrose, 5 ml of 0.025 M manganese chloride, 3 ml of 1% lead nitrate, pH 5.0, filtered with #50 paper) for 1 hour at 37 °C. After washing in Tris-maleate buffer and then sodium cacodylate buffer containing 5% sucrose, sections were briefly treated with 1% sodium sulfide in sodium cacodylate buffer containing 5% sucrose and rinsed well in sodium cacodylate buffer containing 5% sucrose. The sections were then post-fixed in 1% osmium tetroxide and processed for EM embedding.

### Confocal laser scanning microscopy

Immunocytochemistry was performed as previously described^58^. Animals were anesthetized and perfused with Perfusion Fixative Super Reagent (Electron Microscopy Sciences, 1223SK) after being washed with Perfusion Wash Super Reagent (Electron Microscopy Sciences, 1222SK). Brains were dissected and immersed in the same fixative for 24 hours, and then 40-µm sagital sections were made using a vibratome. Brain sections were further stained with indicated antibody overnight and then visualized with Alexa Fluor-conjugated secondary antibody. Imaging was performed using a Plan Apochromat ×20 or ×40/1.4 oil objective lens on a LSM880 laser scanning confocal microscope with the following parameters: eGFP (ex: 488, em: 490–560 with MBS 488), mRFP (ex: 561, em: 582–640 with MBS 458/561), Alexa Fluor 647 (ex: 633, em: 640–710 with MBS 488/561/633) and DAPI (ex: 405, em: 410–483) with best signal scanning model to exclude crosstalk between each wavelength; image acquisition with frame (1,024 × 1,024) scanning mode with averaging 4 line-scan, speed 6. Thio-S staining: Confocal imaged sections were dehydrated and incubated with 1% aqueous Thio-S for 8 minutes. Wash with 80% ethanol (2 × 3 minutes), 95% ethanol (3 minutes) and ddH_2_0 (three times). Analyze slide with the combination of DAPI/eGFP/mRFP filter set. Human AD brain staining: 40-µm free-floating sections cut on a vibratome from fixed tissue blocks were washed once in 1× PBS and rinsed twice in ddH_2_O, followed by incubation in 70% (v:v) formic acid for 12 minutes at 27 °C. Sections were washed 3 × 5 minutes in ddH_2_O and incubated for 4 minutes at 105 °C in 1.0 mM EDTA, pH 8.0, to unmask antigens and allowed to cool to room temperature on a bench, followed by 3 × 5-minute rinse in ddH_2_O. Sections were blocked for 60 minutes in 5% normal horse serum (v:v) and 0.2% Triton X-100 (blocking buffer) and incubated with primary antibodies for 18 hours at 4 °C in blocking buffer, followed by washing 3 × 5 minutes in 1× PBS. Incubation in appropriate secondary antibodies (Invitrogen Alexa Fluor), diluted 1:500 in blocking buffer for 2 hours at 27 °C, was followed by washing 3 × 5 minutes in PBS, and autofluorescence was blocked by autofluorescence blocker (TrueBlack, Biotium) following the manufacturerʼs protocol. Sections were washed 3 × 5 minutes at room temperature and mounted with aqueous medium VectaShield containing DAPI as a nuclear counterstain (Vector Laboratories).

### Vesicle quantification

The same neuronal populations of TRGL single littermate were used as a control. High-resolution images were acquired on a Zeiss LSM880 confocal microscope with Airyscan using a Plan Apochromat ×40/1.4 oil DIC M27 objective. Vesicle quantification analysis was performed as previously described^13^.

### Duolink in situ detection

APP-βCTF was assessed using Duolink II detection reagents orange (Sigma-Aldrich, DUO92013), as instructed by the manufacturer^59^. In brief, cell or brain tissues were incubated overnight at 4 °C in primary APPc (Sigma-Aldrich, A8717, 1:250) and JRF/AβN/25 antibody solution and then washed and incubated in PLA probe plus and minus solution for 1 hour at 37 °C. Tissue sections were washed and incubated with Ligation-Ligase solution for 30 minutes at 37 °C and then incubated with amplification-polymerase solution for 100 minutes at 37 °C. Cell or sections were then mounted using Duolink II and DAPI and viewed using a Zeiss LSM880 confocal microscope.

### Statistics and reproducibility

Statistical parameters, including the definitions and value of sample size (*n*), deviations and *P* values, are reported in the figures and corresponding figure legends. Statistical analyses using Prism 8 (GraphPad) were conducted on data originating from at least three independent experimental replicates. Statistical analyses between two groups were performed by a two-tailed unpaired Student’s *t*-test. Data are expressed as mean ± s.e.m. Differences were considered significant with *P* < 0.05. Depending on the data analysis, sample size related to number of animals was determined by the standards accepted in the field. No specific statistical methods were used to predetermine sample sizes, but sample size was determined based on experience from previous studies^13,60^. Data distribution was assumed to be normal, but this was not formally tested. The samples were not blinded during initial planning because we wanted to ensure that the number of WT and AD mouse models was balanced and age and sex were matched. The mice were then randomly assorted for the studies, and the investigators were blinded when doing the experiments and running data analyses.

### Reporting summary

Further information on research design is available in the Nature Research Reporting Summary linked to this article.

## Online content

Any methods, additional references, Nature Research reporting summaries, source data, extended data, supplementary information, acknowledgements, peer review information; details of author contributions and competing interests; and statements of data and code availability are available at 10.1038/s41593-022-01084-8.

## Supplementary information

Supplementary Information Supplementary Fig. 1 and legend

Reporting Summary

Supplementary Video 1 PANTHOS animated 3D reconstruction.

## Data Availability

Unprocessed scans of all immunoblots and statistical source data in the paper are included as Source Data Figs. 1 and 2, respectively. Correlative light and serial block-face scanning electron microscopy data that support the findings of this study are included as Supplementary Fig. 1 and Movie 1. Other information that supports the findings of this study is available from the corresponding author upon reasonable request. Source data are provided with this paper.

## Source data

Source Data Fig. 2 Statistical Source Data
Source Data Fig. 3 Statistical Source Data
Source Data Fig. 3 Unprocessed western blots
Source Data Fig. 7 Statistical Source Data
Source Data Fig. 7 Unprocessed western blots
Source Data Fig. 8 Statistical Source Data
Source Data Extended Data Fig. 1 Statistical Source Data
Source Data Extended Data Fig. 1 Unprocessed western blots
Source Data Extended Data Fig. 2 Statistical Source Data
Source Data Extended Data Fig. 2 Unprocessed western blots
Source Data Extended Data Fig. 3 Statistical Source Data

## Code availability

No custom software code was used.
