# Supplementary material for: Faulty autolysosome acidification in Alzheimer’s disease mouse models induces autophagic build-up of Aβ in neurons, yielding senile plaques
Source: Nat Neurosci. 2022 Jun 2;25(6):688–701. doi: 10.1038/s41593-022-01084-8 (PMC9174056; doi:10.1038/s41593-022-01084-8)
Supplement: Supplementary file 1 — Supplementary Fig. 1 and legend [file 41593_2022_1084_MOESM1_ESM.pdf]

nature portfolio

https://doi.org/10.1038/s41593-022-01084-8

Supplementary information

Faulty autolysosome acidification in Alzheimer's disease mouse models induces autophagic build-up of Aβ in neurons, yielding senile plaques

In the format provided by the authors and unedited

Lee et al. Supplementary Figure 1

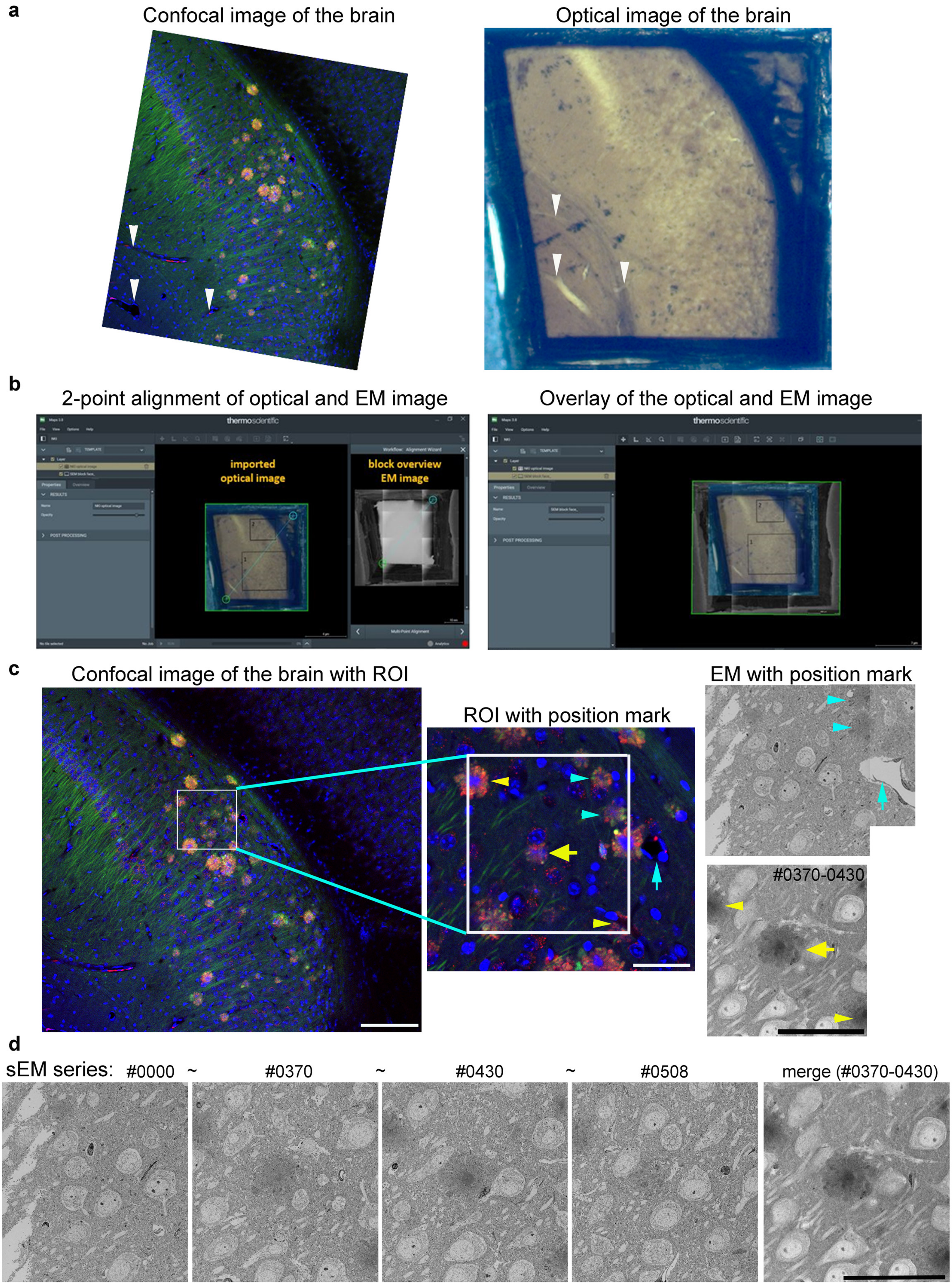

Supplementary Figure 1 | Correlative Light and Serial Block Face Scanning Electron Microscopy method.

a. A confocal image of a 100 μm thick section of the 2.7-month-old 5xFAD/TRGL mouse brain is correlated and overlaid with an optical image of the resin embedded block face of the same section by using capillaries (white arrowheads), which enable the positioning of PANTHOS profiles of interest on the confocal image to the block face (optical image). **b.** Prior to setting up the imaging parameters, the Maps software (Thermo Fisher Scientific, Waltham, USA) was used to perform correlation between the optical image and the EM image to locate the area of interest. Firstly, a block overview EM image was acquired, and an optical image was imported. Secondly, a 2-point alignment strategy was selected where two distinct features were marked on both optical and EM images for the alignment. Lastly, the exact overlay of the optical and EM images allows for precise navigation to the target area where the final data was collected. **c.** The region of interest (ROI) that was imaged using the Apreo Volumescope is highlighted by the white box on the confocal image (left, 10x lens) and enlarged (middle, 20x lens, slightly refocused for getting sharper image) to show landmark features that helped correlate the confocal and EM images. The blue arrowheads show two PANTHOS profiles that are on the top right of the upper EM image. In addition, there is a capillary that is indicated by a blue arrow. The yellow arrowheads indicate two PANTHOS profiles that can be observed on the lower combined EM stack of 60 images (approximately 6 μm) and the PANTHOS profile of interest that is modelled is indicated by the yellow arrow. See also supplementary movie 1. Notice that the high electron density in the center of the profile may represent remnant DAPI signal. Scale bar 100 μm (left panel) and 40 μm (middle, right panel). **d.** The entire data collection covered 509 images with each EM image representing a thickness of 100 nm for a total thickness of 50.9 μm imaged. These panels show various points through the acquisition that covers the PANTHOS profile of interest with the panel on the right showing 60 images that have been combined to show part of the profile. Scale bar 40 μm.
