# Supplementary material for: Faulty autolysosome acidification in Alzheimer’s disease mouse models induces autophagic build-up of Aβ in neurons, yielding senile plaques
Source: Nat Neurosci. 2022 Jun 2;25(6):688–701. doi: 10.1038/s41593-022-01084-8 (PMC9174056; doi:10.1038/s41593-022-01084-8)
Supplement: Supplementary file 2 — Reporting Summary [file 41593_2022_1084_MOESM2_ESM.pdf]

nature portfolio

Corresponding author(s): JU-HYUN LEE and RALPH A. NIXON

Last updated by author(s): Apr 19, 2022

Reporting Summary

Nature Portfolio wishes to improve the reproducibility of the work that we publish. This form provides structure for consistency and transparency in reporting. For further information on Nature Portfolio policies, see our Editorial Policies and the Editorial Policy Checklist.

Statistics

For all statistical analyses, confirm that the following items are present in the figure legend, table legend, main text, or Methods section.

n/a	Confirmed	
☐	☒	The exact sample size (n) for each experimental group/condition, given as a discrete number and unit of measurement
☐	☒	A statement on whether measurements were taken from distinct samples or whether the same sample was measured repeatedly
☐	☒	The statistical test(s) used AND whether they are one- or two-sided<br>*Only common tests should be described solely by name; describe more complex techniques in the Methods section.*
☐	☒	A description of all covariates tested
☐	☒	A description of any assumptions or corrections, such as tests of normality and adjustment for multiple comparisons
☐	☒	A full description of the statistical parameters including central tendency (e.g. means) or other basic estimates (e.g. regression coefficient) AND variation (e.g. standard deviation) or associated estimates of uncertainty (e.g. confidence intervals)
☐	☒	For null hypothesis testing, the test statistic (e.g. F, t, r) with confidence intervals, effect sizes, degrees of freedom and P value noted<br>*Give P values as exact values whenever suitable.*
☒	☐	For Bayesian analysis, information on the choice of priors and Markov chain Monte Carlo settings
☐	☒	For hierarchical and complex designs, identification of the appropriate level for tests and full reporting of outcomes
☒	☐	Estimates of effect sizes (e.g. Cohen's d, Pearson's r), indicating how they were calculated

Our web collection on statistics for biologists contains articles on many of the points above.

Software and code

Policy information about availability of computer code

Data collection	Confocal images were acquired using a LSM 880 Confocal microscope (Zeiss) and the ZEN software package (Zen black 2.1 SP3, Zeiss).<br>SEM images were acquires using an Apreo scaning electron microscope (ThermoFisher) and Maps software (v3.4, ThermoFisher).
Data analysis	Histological analysis was performed in a blind fashion. Confocal image analysis for Hue angle was done with Zen blue v2.1.57.1000, Zeiss). SEM images analysis was done with Maps software (v3.4, ThermoFisher). Data analysis was done with Prism (version 8.1.0, GraphPad Software, Inc). Data expresses as mean±S.E.M. Two group comparisons were analyzed by the two-tailed t-test otherwise by ANOVA test. Difference were considered stastically significant for probability values less than 0.05.

For manuscripts utilizing custom algorithms or software that are central to the research but not yet described in published literature, software must be made available to editors and reviewers. We strongly encourage code deposition in a community repository (e.g. GitHub). See the Nature Portfolio guidelines for submitting code & software for further information.

Data

Policy information about availability of data

All manuscripts must include a data availability statement. This statement should provide the following information, where applicable:

- Accession codes, unique identifiers, or web links for publicly available datasets
- A description of any restrictions on data availability
- For clinical datasets or third party data, please ensure that the statement adheres to our policy

Unprocessed scans of all immunoblots and statistical source data in the paper are included as Source Data figure 1 and 2, respectively. Correlative Light and serial Block Face Scanning Electron microscope data that support the finding of this study is included as Supplementary Figure 1 and movie 1. Other information that supports the findings of this study is available from the corresponding author upon request.

Field-specific reporting

Please select the one below that is the best fit for your research. If you are not sure, read the appropriate sections before making your selection.

☒ Life sciences ☐ Behavioural & social sciences ☐ Ecological, evolutionary & environmental sciences

For a reference copy of the document with all sections, see nature.com/documents/nr-reporting-summary-flat.pdf

Life sciences study design

All studies must disclose on these points even when the disclosure is negative.

Sample size	Depending on the data analysis, sample size related to number of animals was determined by the standards accepted in the field. No specific statistical methods were used to pre-determine sample sizes but sample size was determined based on experience from previous studies (Lee et al. Autophagy 2019, Meyer-Luehmann et al. Nature 2008).
Data exclusions	No data were excluded.
Replication	The number of biological or technical replicates for each experimental group is listed in the corresponding figure legends. All image analyses were sampled across at least 3 animals. vATPase analysis were sampled across 4 replicates from at least 3 animals. AV fractions were sampled across 2 replicates from the pool of at least 5 animals. Biochemical analyses were sampled across 2 replicates from at least 3 animals. Human AD brain analyses were sampled from 3 individuals. Analysis was performed independently by multiple investigators to ensure reproducibility. All attempts at replication were successful.
Randomization	For all experiments, mice were randomly allocated into each experimental group by P.S and J.P. The order of animals was randomized for each experiment to minimize potential effects from given imaging session or staining cohort.
Blinding	The samples were not blinded during initial planning of animal selection because we wanted to ensure that the number of wild-type and AD mouse models were balanced and, age and sex were matched. The mice were then randomly assorted for the studies and the investigators were blinded when doing the experiments and running data analyses.

Reporting for specific materials, systems and methods

We require information from authors about some types of materials, experimental systems and methods used in many studies. Here, indicate whether each material, system or method listed is relevant to your study. If you are not sure if a list item applies to your research, read the appropriate section before selecting a response.

Materials & experimental systems

n/a	Involved in the study	
☐	☒	Antibodies
☐	☒	Eukaryotic cell lines
☒	☐	Palaeontology and archaeology
☐	☒	Animals and other organisms
☒	☐	Human research participants
☒	☐	Clinical data
☒	☐	Dual use research of concern

Methods

n/a	Involved in the study	
☒	☐	ChIP-seq
☒	☐	Flow cytometry
☒	☐	MRI-based neuroimaging

Antibodies

Antibodies used	Anti-PS1 loop mouse mAb (MAB5232:clone PS1-loop, 1/1000) and anti-nicastrin mouse mAb (MAB5556: clone 9C3, 1/1000) were purchased from Chemicon. Rabbit anti-CTSD (Rudy4, 1/2000) antibody and NFL (21.4, 1/250) were produced in house 13. CTSB from Neuromics (GT15047, 1/250). LAMP2 from DSHB (ABL-93, 1/200). LIMP2 from Novus (NB400-129, 1/200). Antibodies directed against APP, Aβ and/or other APP proteolytic species included: APPc (Sigma, A8717, 1/250); 4G8 (BioLegend: clone 4G8, 800701, 1/250); C1/6.1 monoclonal antibody against the C-terminal 20 residues of APP (made in-house, 1/400) (Nathan Kline Institute, USA)); and additional mouse monoclonal antibodies were generous gift from Dr. Marc Mercken (Janssen Pharmaceutica/Johnson & Johnson, Belgium): JRF/AβN/25 (specific to Aβ1-7, 1/200); 3D6 (specific to Aβ1-5, 1/250); JRF/cAb42/26 (specific to Aβ42, 1/200) 85. MAP2 (Sigma, M9942: clone HM-2, 1/250). NSE (DAKO, M0873: clone BBS/NC/VI-H14, 1/250). Histone H3 (4499: clone D1H2, 1/200). Lamin A/C (4777: clone 4C11, 1/200) and Tom20 (42406: clone D8T4N, 1/2000) were from Cell Signaling. KDM1/LSD1 (Abcam, ab129195: clone EPR6825). GFAP (Sigma, AB5804, 1/250), Ibal (Wako, 019-19741, 1/250). ATP6 V1A (GenneTex, GTX110815, 1/1000), ATP6 V0a1 (Abcam, ab176858, 1/2000), Rab5 (Abcam, ab218624: clone EPR21801, 1/1000). Rab7 (Cell Signaling, 9367: clone D95F2, 1/1000). PDI (BD Science, 610946: clone 34, 1/1000), STX6 (Cell Signaling, 2869: clone C34B2, 1/2000), Tubulin (Sigma, T8535:clone JDR.3B8, 1/5000), Actin (Sigma, A1978: clone AC-15, 1/5000), anti-p62 (ProgenBiotechnk, GP62-C, 1/500). Anti-SEC61B rabbit pAb (15087-1-AP, 1/1000) was from Proteintech. HRP- linked Rabbit IgG (711-035-152, 1/5000), Mouse IgG (711-035-150, 1/5000), Rat IgG (712-035-150), and Goat IgG (705-035-003) secondary antibodies were purchased from Jackson ImmunoResearch. Prolong Diamond Antifade Mount (P36961), Goat anti-Mouse Alexa 647 (A21235), Goat anti-Rat Alexa 647 (A21247), Goat anti-

Rabbit (A21245) Alexafluor 647, and Donkey anti-Rabbit Alexa 405 (A48254) secondary antibodies were from ThermoFisher. Mouse on Mouse (M.O.M) detection kit (BMK-2201), normal-donkey (S-2000-20) and normal-goat (S-100) serum blocking solution were from Vector Lab. Thioflavin-S (T1892) form Sigma-Aldrich.

Validation

Commercial antibodies were validated by the manufacturer (See below link).
Anti-PS1 Loop (https://www.emdmillipore.com/US/en/product/Anti-Presenilin-1-Antibody-loop-a.a.-263-378-CT-clone-PS1-loop,MM_NF-MAB5232).
Anti-Nicastrin (https://www.emdmillipore.com/US/en/product/Anti-Nicastrin-Antibody,MM_NF-MAB5556).
Anti-CTSB (https://www.neuromics.com/ittrium/reference/D8x13dfx8x1).
Anti-LAMP2 (https://dshb.biology.uiowa.edu/ABL-93).
Anti-LIMP2 (https://www.novusbio.com/products/limpii-sr-b2-antibody_nb400-129).
Anti-APP C-terminal (https://www.sigmaaldrich.com/US/en/product/sigma/a8717).
4G8 (https://www.biolegend.com/en-us/search-results/anti-beta-amyloid-17-24-antibody-10999).
Mur monoclonal Anti-MAP2 (https://www.sigmaaldrich.com/US/en/product/sigma/m9942).
Anti-NSE (https://www.agilent.com/en/product/immunohistochemistry/antibodies-controls/primary-antibodies/neuron-specific-enolase-(concentrate)-76548#specifications).
Anti-Histone H3 (https://www.cellsignal.com/products/primary-antibodies/histone-h3-d1h2-xp-rabbit-mab/4499).
Anti-Lamin A/C (https://www.cellsignal.com/products/primary-antibodies/lamin-a-c-4c11-mouse-mab/4777).
Anti-Tom20 (https://www.cellsignal.com/products/primary-antibodies/tom20-d8t4n-rabbit-mab/42406).
Anti-KDM/LSD1 (https://www.abcam.com/products?keywords=KDM1%2FLSD1&selected.classification=Primary+antibodies).
Anti-GFAP (https://www.sigmaaldrich.com/US/en/product/mm/ab5804).
Anti-Iba1 (https://labchem-wako.fujifilm.com/us/product/detail/W01W0101-1974.html)..
Anti-ATP6 V0a1 (https://www.abcam.com/atp6v0a1-antibody-ab176858.html).
Anti-Rab5 (https://www.abcam.com/rab5-antibody-epr21801-ab218624.html)
Anti-Rab7 (https://www.cellsignal.com/products/primary-antibodies/rab7-d95f2-xp-rabbit-mab/9367).
Anti-PDI (https://www.bdbiosciences.com/en-us/products/reagents/microscopy-imaging-reagents/immunofluorescence-reagents/purified-mouse-anti-pdi.610946).
Anti-Stx6 (https://www.cellsignal.com/products/primary-antibodies/syntaxin-6-c34b2-rabbit-mab/2869).
Anti-bTubulin I+II (https://www.sigmaaldrich.com/US/en/search/t8535).
Anti-b-Actin (https://www.sigmaaldrich.com/US/en/search/a1978).
Anti-p62 C-terminal (Sqstm1) (https://us.progen.com/anti-p62-SQSTM1-C-terminus-guinea-pig-polyclonal-serum/GP62-C)..
Anti-Sec1b (https://www.ptglab.com/products/SEC61B-Antibody-15087-1-AP.htm).

Rabbit polyclonal anti-CTSD was validated by our study: Lee, J.-H., Yu, W.H., Kumar, A., Lee, S., Mohan, P.S., Peterhoff, C.M., Wolfe, D.M., Martinez-Vicente, M., Massey, A.C., Sovak, G., et al. (2010). Lysosomal proteolysis and autophagy require presenilin 1 and are disrupted by Alzheimer-related PS1 mutations. Cell 141, 1146-1158.

Mouse monoclonal anti-NFL was validated by our study: Yuan, A., Sershen, H., Veeranna, Basavarajappa, B.S., Kumar, A., Hashim, A., Berg, M., Lee, J.H., Sato, Y., Rao, M.V., et al. (2015). Neurofilament subunits are integral components of synapses and modulate neurotransmission and behavior in vivo. Mol Psychiatry 20, 986-994.

JRF/AβN/25 was validated by our study: Mathews, P.M., Jiang, Y., Schmidt, S.D., Grbovic, O.M., Mercken, M., and Nixon, R.A. (2002). Calpain activity regulates the cell surface distribution of amyloid precursor protein. Inhibition of calpains enhances endosomal generation of beta-cleaved C-terminal APP fragments. J Biol Chem 277, 36415-36424.

JRF/cAb42/26 was validated by this study: Janus, C. et al. Aβ peptide immunization reduces behavioural impairment and plaques in a model of Alzheimer's disease. Nature 408, 979-982 (2000).

C1/6.1 was validated by our study: Jiang, Y., Mullaney, K.A., Peterhoff, C.M., Che, S., Schmidt, S.D., Boyer-Boiteau, A., Ginsberg, S.D., Cataldo, A.M., Mathews, P.M., and Nixon, R.A. (2010). Alzheimer's-related endosome dysfunction in Down syndrome is Aβ-independent but requires APP and is reversed by BACE-1 inhibition. Proc Natl Acad Sci U S A, 1630-1635.

Eukaryotic cell lines

Policy information about cell lines

Cell line source(s)

Neuro-2a (N2a) cells from ATCC (https://www.atcc.org/products/ccl-131) and N2a APPswe cells were generous gift from Dr. Gopal Thinakaran (Morsani College of Medicine, University of South Florida). Thinakaran G, Teplow DB, Siman R, Greenberg B, Sisodia SS. Metabolism of the ``Swedish'' Amyloid Precursor Protein Variant in Neuro2a (N2a) Cells. J Biol Chem. 1996;271:9390-7.

Authentication

Cells were validated by short tandem repeat analysis

Mycoplasma contamination

Cells were routinely tested for mycoplasma and all cell lines tested negative for mycoplasma contamination.

Commonly misidentified lines
(See ICLAC register)

No commonly misidentified cell lines were used

Animals and other organisms

Policy information about studies involving animals; ARRIVE guidelines recommended for reporting animal research

Laboratory animals	The mice were maintained in the Nathan Kline Institute (NKI) animal facility and housed at ~22.8˚C room temperature with a humidity level of ~55 % and 12 hrs light/dark cycle. All animal experiments were performed according to “Principles of Animal Care” and approved by the Institutional Animal Care and Use Committee (IACUC) at the NKI.<br><br>Mouse Tg2576 B6;Dba/2F1;SW (Holcomb et al., 1998): 6 and 12 months old male together with age matched WT littermates were used.<br>Mouse Tg2576, B6;Dba/2F1;SW (Holcomb et al., 1998)/TRGL: 1.6, 5, 6, 10 and 12 months old female together with age matched TRGL(-/+) littermates were used.<br>Mouse 5XFAD, C57BL/6NTAC (Kimura and Ohno, 2009)/TRGL: 1.6, 2.7, 4, 5, and 6 months old female together with gae matched TRGL(-/+) littermates were used.<br>Mouse TgCRND8, 129X1/SvJ (129X1) (Yang et al., 2011)/TRGL: 1.9 months old male together with age matched TRGL(-/+) littermates were used<br>Mouse PS/APP, B6;Dba/2F1;SW (Cataldo et al., 2004)/TRGL: 3.1 months old male together with age matched TRGL6(-/+) littermates were used.<br>Mouse APP51, B6 (Bodendorf et al., 2002): 30 months old female together with age matched WT littermates were used.<br>Mouse APP51, B6 (Bodendorf et al., 2002)/TRGL: 12, 15, 20, 25.5, and 28 months old female together with age matched TRGL(-/+) littermates were used.<br>Mouse TRGL, B6. (Lee et al., 2019): 1.6, 1.9, 2.7, 3.1, 4, 5, 6, 10, 12, 20, 25.5, and 28 months old male or female TRGL(-/+) were used.<br><br>References<br><br>Bodendorf, U., Danner, S., Fischer, F., Stefani, M., Sturchler-Pierrat, C., Wiederhold, K.-H., Staufenbiel, M., and Paganetti, P. (2002). Expression of human β-secretase in the mouse brain increases the steady-state level of β-amyloid. Journal of Neurochemistry 80, 799-806.<br>Cataldo, A.M., Peterhoff, C.M., Schmidt, S.D., Terio, N.B., Duff, K., Beard, M., Mathews, P.M., and Nixon, R.A. (2004). Presenilin mutations in familial Alzheimer disease and transgenic mouse models accelerate neuronal lysosomal pathology. J Neuropathol Exp Neurol 63, 821-830.<br>Holcomb, L., Gordon, M.N., McGowan, E., Yu, X., Benkovic, S., Jantzen, P., Wright, K., Saad, I., Mueller, R., Morgan, D., et al. (1998). Accelerated Alzheimer-type phenotype in transgenic mice carrying both mutant amyloid precursor protein and presenilin 1 transgenes. Nat Med 4, 97-100.<br>Kimura, R., and Ohno, M. (2009). Impairments in remote memory stabilization precede hippocampal synaptic and cognitive failures in 5XFAD Alzheimer mouse model. Neurobiol Dis 33, 229-235.<br>Lee, J.-H., Rao, M.V., Yang, D.-S., Stavrides, P., Im, E., Pensalfini, A., Huo, C., Sarkar, P., Yoshimori, T., and Nixon, R.A. (2019). Transgenic expression of a ratiometric autophagy probe specifically in neurons enables the interrogation of brain autophagy in vivo. Autophagy 15, 543-557.<br>Yang, D.S., Stavrides, P., Mohan, P.S., Kaushik, S., Kumar, A., Ohno, M., Schmidt, S.D., Wesson, D., Bandyopadhyay, U., Jiang, Y., et al. (2011). Reversal of autophagy dysfunction in the TgCRND8 mouse model of Alzheimer's disease ameliorates amyloid pathologies and memory deficits. Brain 134, 258-277.
Wild animals	Wilds animals were not investigated in the study.
Field-collected samples	Field-collected samples were not studied in the study.
Ethics oversight	All procedures were approved by the institutional Animal Care and Use Committee of Nathan S. Kline Institute.

Note that full information on the approval of the study protocol must also be provided in the manuscript.
