# Supplementary figures and images for: Faulty autolysosome acidification in Alzheimer’s disease mouse models induces autophagic build-up of Aβ in neurons, yielding senile plaques
Source: Nat Neurosci. 2022 Jun 2;25(6):688–701. doi: 10.1038/s41593-022-01084-8 (PMC9174056; doi:10.1038/s41593-022-01084-8)

Figure 3c

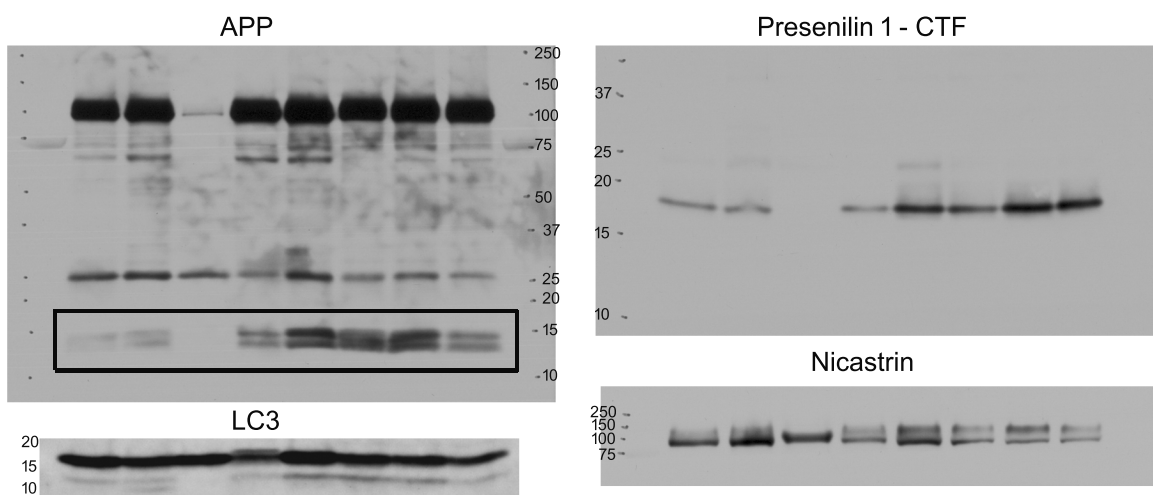

Supplement: Source Data Fig. 3 — Unprocessed western blots [file 41593_2022_1084_MOESM6_ESM.pdf]

Figure 7e

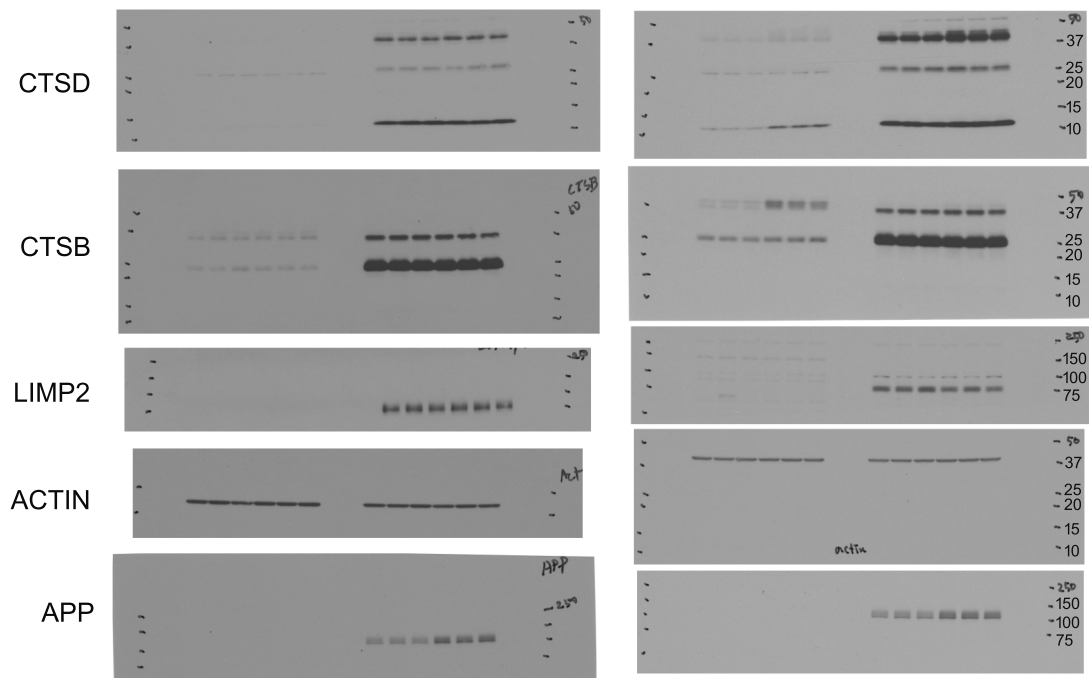

Supplement: Source Data Fig. 7 — Unprocessed western blots [file 41593_2022_1084_MOESM8_ESM.pdf]

Extended Figure 1c

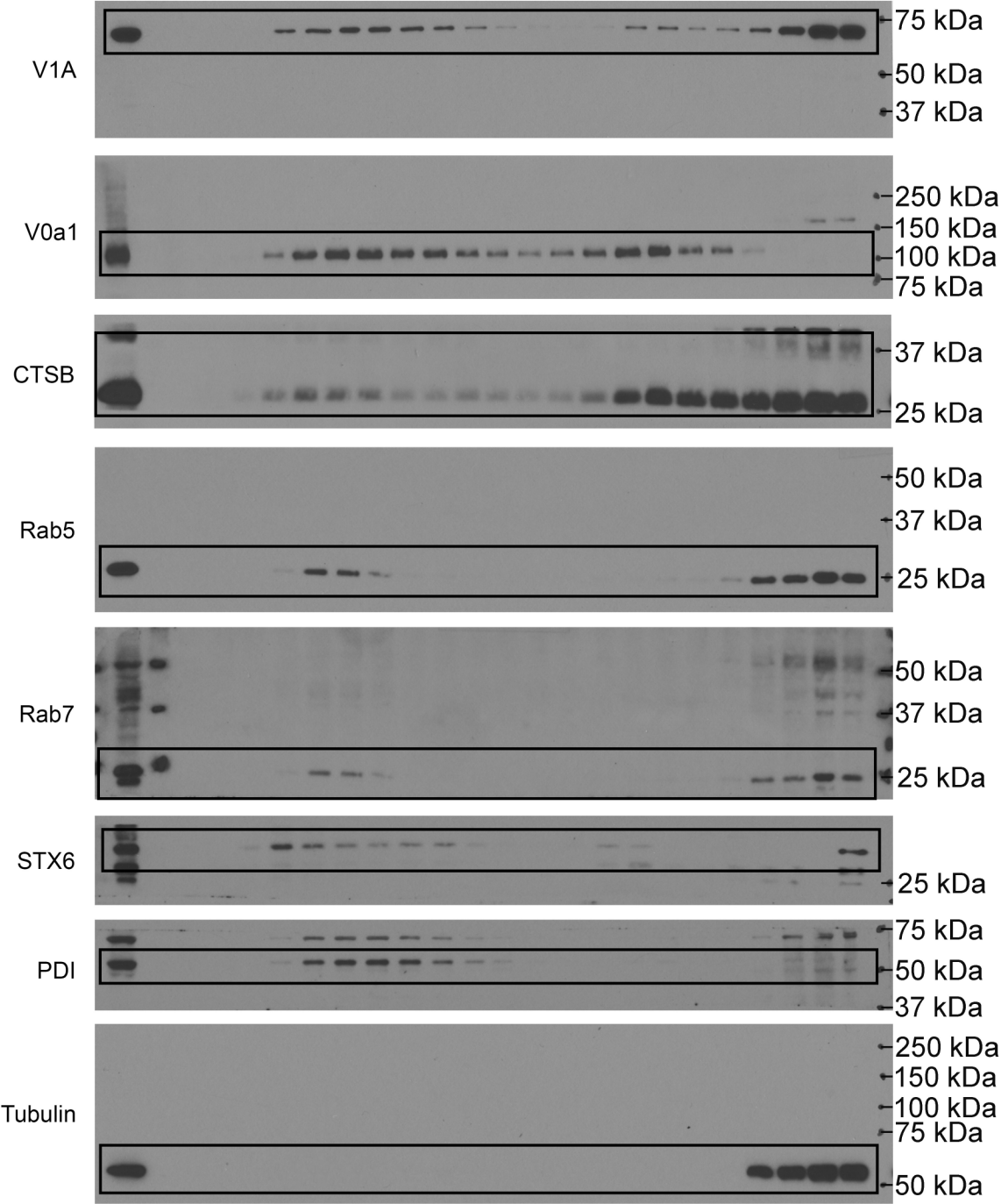

Supplement: Source Data Extended Data Fig. 1 — Unprocessed western blots [file 41593_2022_1084_MOESM11_ESM.pdf]

Extended Data Figure 2a

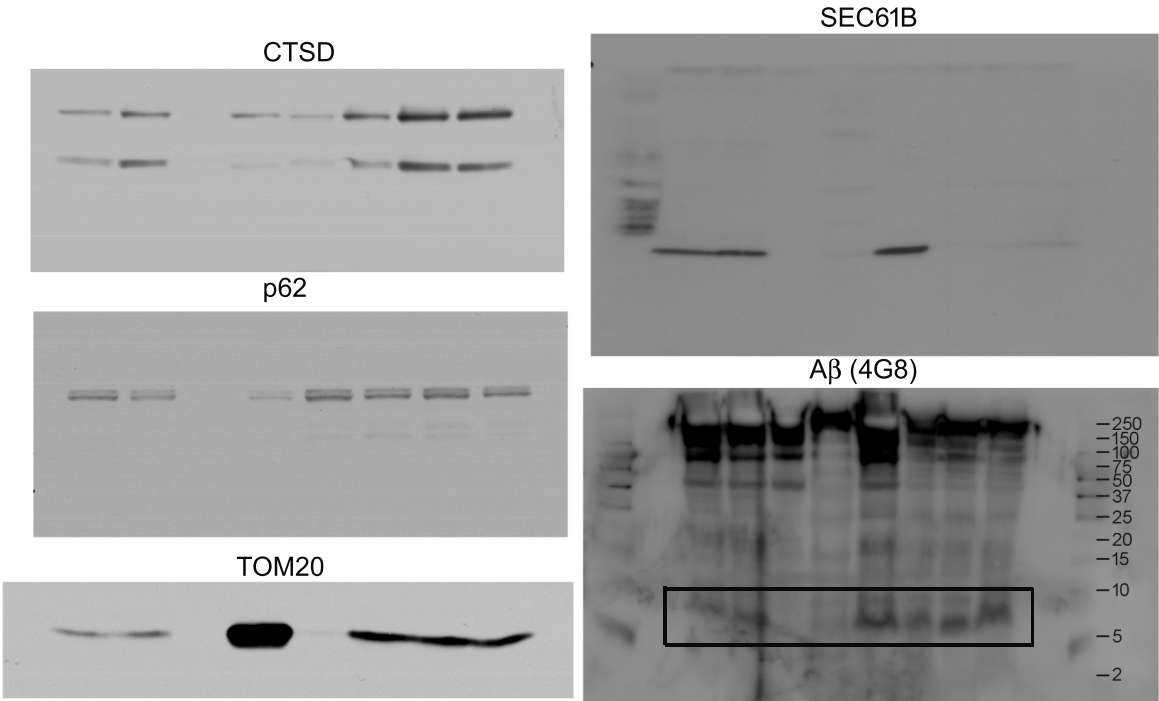

Supplement: Source Data Extended Data Fig. 2 — Unprocessed western blots [file 41593_2022_1084_MOESM13_ESM.pdf]
